# Residual Complex I activity and amphidirectional Complex II operation support glutamate catabolism through mtSLP in anoxia

**DOI:** 10.1038/s41598-024-51365-4

**Published:** 2024-01-19

**Authors:** Dora Ravasz, David Bui, Sara Nazarian, Gergely Pallag, Noemi Karnok, Jennie Roberts, Bryan P. Marzullo, Daniel A. Tennant, Bennett Greenwood, Alex Kitayev, Collin Hill, Timea Komlódi, Carolina Doerrier, Kristyna Cunatova, Erika Fernandez-Vizarra, Erich Gnaiger, Michael A. Kiebish, Alexandra Raska, Krasimir Kolev, Bence Czumbel, Niven R. Narain, Thomas N. Seyfried, Christos Chinopoulos

**Affiliations:** 1 Department of Biochemistry, Semmelweis University, Budapest, 1094 Hungary; 2 Institute of Metabolism and Systems Research, College of Medical and Dental Sciences, University of Birmingham, Birmingham, B15 2TT UK; 3 BERG, Framingham, MA 01701 USA; 4 Oroboros Instruments, Innsbruck, Austria; 5 Department of Biomedical Sciences, University of Padova, 35131 Padova, Italy; 6 Biology Department, Boston College, Chestnut Hill, Boston, MA 02467 USA

**Keywords:** Biochemistry, Metabolomics

## Abstract

Anoxia halts oxidative phosphorylation (OXPHOS) causing an accumulation of reduced compounds in the mitochondrial matrix which impedes dehydrogenases. By simultaneously measuring oxygen concentration, NADH autofluorescence, mitochondrial membrane potential and ubiquinone reduction extent in isolated mitochondria in real-time, we demonstrate that Complex I utilized endogenous quinones to oxidize NADH under acute anoxia. ^13^C metabolic tracing or untargeted analysis of metabolites extracted during anoxia in the presence or absence of site-specific inhibitors of the electron transfer system showed that NAD^+^ regenerated by Complex I is reduced by the 2-oxoglutarate dehydrogenase Complex yielding succinyl-CoA supporting mitochondrial substrate-level phosphorylation (mtSLP), releasing succinate. Complex II operated amphidirectionally during the anoxic event, providing quinones to Complex I and reducing fumarate to succinate. Our results highlight the importance of quinone provision to Complex I oxidizing NADH maintaining glutamate catabolism and mtSLP in the absence of OXPHOS.

## Introduction

All mammalian mitochondria harbor Complex I (CI, NADH:ubiquinone oxidoreductase, EC 1.6.5.3). CI catalyzes the oxidation of matrix NADH by ubiquinone (UQ) to produce NAD^+^ and ubiquinol (UQH_2_), which is coupled to the translocation of four H^+^ across the mitochondrial inner membrane and the transfer of electrons downstream to FeS clusters^1^. Mindful of the limitations in quantifying NADH oxidation by CI^2^ and the enhancement of NADH autofluorescence by mere binding to CI^3^, it is difficult to decipher the extent of the contribution of CI in altering the mitochondrial NADH/NAD^+^ ratio. Nevertheless, it is a textbook definition that cessation of the electron transfer system (ETS) due to lack of oxygen, pharmacological inhibition or genetic ablation of respiratory components leads to an accumulation of reduced compounds in the mitochondrial matrix, exactly because CI is not able to oxidize NADH. In turn, this increase in matrix NADH/NAD^+^ ratio is expected to impair the function of matrix dehydrogenases, preventing mitochondria from catabolizing substrates in the citric acid cycle^4^. Having said that, the catabolism of glutamine through oxidative decarboxylation of 2-oxoglutarate (oxoglutarate, Og, α-ketoglutarate) during hypoxia has been unequivocally demonstrated^5^. The question arises, what provides NAD^+^ to 2-oxoglutarate dehydrogenase complex (OgDHC) in hypoxia? Using a battery of untargeted and targeted ^13^C metabolic tracer analyses, biochemical, electrochemical and fluorescence assays, we report that in isolated murine mitochondria experiencing acute true anoxia or in the presence of cyanide, the residual activity of CI is sufficient to provide NAD^+^ to OgDHC and maintain the oxidative decarboxylation branch of 2-oxoglutarate supporting mitochondrial substrate-level phosphorylation (mtSLP). We further show that during the anoxic event Complex II (CII, succinate:ubiquinone oxidoreductase, EC 1.3.99.1) operates amphidirectionally, providing quinones to Complex I and reducing fumarate to succinate.

## Materials and methods

All methods were carried out in accordance with relevant guidelines and regulations of the Semmelweis University.

All methods reported are in accordance with the ARRIVE guidelines (https://arriveguidelines.org).

### Experimental model and subject details

#### Animals

Mice were of C57Bl/6N or 6J (the latter only for the right panels of main figure 6 and supplementary figure panels 8K and 8L) or 129/Sv (for main figure 6, left panels) background. The animals used in our study were of either sex and between 2 and 6 months of age. Mice were housed in a room maintained at 20–22 °C on a 12-h light–dark cycle with food and water available ad libitum, unless otherwise indicated. For preparation of mitochondria from their organs, mice were killed by cervical dislocation without euthanasia. The study was approved by the Animal Care and Use Committee of the Semmelweis University (*Egyetemi Állatkísérleti Bizottság*, protocol code F16-00177 [A5753-01]; date of approval: May 15, 2017). All procedures regarding animals complied with the ARRIVE guidelines.

#### Mitochondrial isolation

Liver and heart mitochondria from all animals were isolated as described in^6^ with minor modifications. Mice were killed by cervical dislocation without euthanasia. The organs were rapidly removed and immediately placed in ice-cold isolation buffer, the composition of which was, for liver: 225 mM mannitol, 75 mM sucrose, 5 mM HEPES (free acid), 1 mM EGTA and 1 mg/ml bovine serum albumin (fatty acid-free), with the pH adjusted to 7.4 with Trizma®, and for heart: 100 mM KCl, 50 mM Trizma, 5 mM MgCl_2_, 2 mM EGTA and 2.5 mg/ml bovine serum albumin (fatty acid-free), with the pH adjusted to 7.4 with HCl, to which 1 mM ATP was just freshly added. Next, the organs were chopped, washed, homogenized and the homogenate was centrifuged at 1,250 g for 10 min. The upper fatty layer of the centrifuged homogenate was aspirated and the pellet was discarded. The remaining supernatant was centrifuged at 10,000 g for 10 min. After this step the supernatant was discarded and the pellet was resuspended in isolation buffer, and centrifuged again at 10,000 *g* for 10 min. At the end the pellet was resuspended in 0.2 ml of a buffer with the same composition as the liver isolation buffer but with 0.1 mM EGTA. Non-synaptic brain mitochondria were isolated on a Percoll gradient as described previously^7^ , with minor modifications detailed in^8^. Protein concentration was determined using the bicinchoninic acid assay, and calibrated using bovine serum standards^9^ using a Tecan Infinite® 200 PRO series plate reader (Tecan Deutschland GmbH, Crailsheim, Germany).

#### Determination of membrane potential (ΔΨ_mt_)

Δ*Ψ*_mt_ of isolated mitochondria (0.5–1 mg mitochondria in 2 mL buffer medium, the composition of which was 8 mM KCl, 110 mM K-gluconate, 10 mM NaCl, 10 mM Hepes, 10 mM KH_2_PO_4_, 0.01 mM EGTA, 10 mM mannitol, 1 mM MgCl_2_, 0.5 mg/ml bovine serum albumin (fatty acid-free), pH 7.25, containing substrates as indicated in the figure legends) was estimated fluorimetrically with 5 µM rhodamine 123^10^. Fluorescence was recorded using the NextGen-O2k prototype equipped with the O2k-Fluo Smart Module, with optical sensors including a LED (465 nm; < 505 nm short-pass excitation filter), a photodiode and specific optical filters (> 560 nm long-pass emission filter)^11^. Experiments were performed at 37  °C.

#### Mitochondrial respiration

Oxygen consumption was monitored polarographically using an Oxygraph-2k. 0.5–1 mg of mitochondria were suspended in 2 ml incubation medium, the composition of which was identical to that for Δ*Ψ*_mt_ determination. Experiments were performed at 37  °C. Oxygen concentration (µM) and oxygen flux (pmol·s ^−1^·mg ^−1^; negative time derivative of oxygen concentration, divided by mitochondrial mass per volume and corrected for instrumental background oxygen flux arising from oxygen consumption of the oxygen sensor and back-diffusion into the chamber) were recorded using DatLab software (Oroboros Instruments).

#### Determination of NADH autofluorescence

NADH autofluorescence was measured using the NADH-Module of the NextGen-O2k (Oroboros Instruments) or a Hitachi F-7000 fluorescence spectrophotometer, as indicated in the text. The NextGen-O2k allows simultaneous measurement of oxygen consumption and NADH autofluorescence, incorporating an ultraviolet (UV) LED with an excitation wavelength of 365 nm and an integrated spectrometer which records a wavelength range between 450 and 590 nm. The light intensity of the LED was set to 10 mA. 0.5–1 mg of mitochondria were suspended in 2 mL incubation medium, the composition of which was identical to that for Δ*Ψ*_mt_ determination, as described in^12^. Experiments were performed at 37 °C.

#### NADH alkaline extraction and quantification

NADH was extracted and quantified in isolated mouse liver mitochondria as described in^13^, with minor modifications. Briefly, 2 mg mitochondria in 2 ml were incubated as described above under *mitochondrial respiration*. Reactions were stopped by the addition of 50 µl 52.9 wt% KOH in water, and 50 µl ethanol was added to 100 µl samples. After 30 min the solution was neutralized with a 0.4 M Tris, 0.6 M KH_2_PO_4_ mixture and left to flocculate for 10 min. The suspension was centrifuged and 2 µl 1 M pyruvate was added to 200 µl supernatant, oxidation of NADH was measured by the decrease in fluorescence (340 nm Ex., 435 nm Em.) upon the addition of 1 µl 96.8 U/ml bovine H-type LDH in a white 96 well plate. Fluorescence was calibrated by standard addition of 1 µl 0.1 mM NADH. Each measurement was done with technical triplicates.

#### Mitochondrial Q redox state

Coenzyme UQ redox state of isolated mitochondria suspended in a buffer composition identical to that for Δ*Ψ*_mt_ determination was followed amperometically using a three electrode system described in^14,15^ but with coenzyme Q_2_ (CoQ_2_, 1 µM) as mediator, using the Q-Module of the NextGen-O2k^16,17^. The reference electrode was Ag/AgCl/(3M KCl). The auxiliary electrode was made of platinum and the working electrode was fabricated from glassy carbon. The three electrode system has been validated in^16,17^. Oxidation peak potential of CoQ_2_ measured by cyclic voltammetry was set to the glassy carbon to measure the oxidation of reduced CoQ_2_. UQ redox state was recorded simultaneously with O_2_ flux and rhodamine 123 fluorescence. Arbitrarily, we assigned the effect of ADP and anoxia as the 100% and 0% internal reference points of UQ oxidized state scale, respectively. All drugs used in this study were verified not to exert any artefactual alterations on the electrodes of the Q module using cyclic voltammetry, shown in supplementary Fig. 17.

#### Mitochondrial swelling

Swelling of isolated mitochondria was assessed by measuring light scatter at 520 nm (37 °C) in a Hitachi F-7000 fluorescence spectrophotometer. 0.5 mg of mouse liver mitochondria were suspended in 2 ml incubation medium, the composition of which was identical to that for Δ*Ψ*_mt_ determination. Experiments were performed at 37 °C. Anoxic conditions were achieved by manufacturing a custom-made plug for polymethacrylate cuvettes by 3D-printing. The plug 3D design and instructions for use are published in https://www.thingiverse.com/thing:3156148. At the end of each experiment, the non-selective pore-forming peptide alamethicin (40 μg) was added as a calibration standard to cause maximal swelling.

#### Prothrombin time measurement

Blood samples were taken from the saphenous vein in 110 mM Na_3_-citrate (citrate/blood volume ratio 1:10), as described in^18^. After centrifugation at 2500*g* for 15 min the plasma supernatant was collected and used for the measurement within 4 h. Prothrombin time was measured with Technoplastin-HIS reagent (Technoclone Herstellung von Diagnostika und Arzneimitteln GmbH, Vienna, Austria) in a coagulometer KC-1A (Amelung, Lemgo, Germany) as the time to form clots from 100 µl plasma diluted twofold in 10 mM HEPES 150 mM NaCl pH 7.4 buffer by 200 µl Technoplastin-HIS reagent.

#### Untargeted metabolite analysis

Mitochondrial suspensions were ’spiked’ with 1 mM L-norleucine, a non-metabolizable substrate that yields a highly recognizable signature during the metabolite analysis and was used for normalizing volumes and keeping pipetting errors at check. At specified times during the experiments (indicated in the text) three 0.6 mL aliquots from the 2 mL mitochondrial suspensions were spun at 14,000 rpm for 5 min at 4 °C. Supernatants (0.5 mL) were separated from the pellets. Any remaining visible aqueous phase was removed from the pellets and discarded. Both pellets and supernatants were transferred to new tubes and to each tube 0.5 mL of ice-cold 80% methanol was added. The tubes were snap frozen in liquid nitrogen during processing to ensure samples were kept cold and transferred to − 80 °C until further processing. Approximately two days later, samples were sonicated for 10 min at room temperature and then centrifuged at 14,000 rpm for 10 min at 4 °C. All of the supernatants were removed, transferred to Eppendorf tubes and evaporated to dryness overnight using a centrifugal evaporator. Once dry, the dried lysates were stored at − 80 °C until further analysis. Dried lysates were reconstituted in 2:1:1 acetonitrile:MeOH:H_2_O, to yield a concentration of 200 mg/mL and spun at 14,000 rpm for 10 min at 4 °C to remove excess debris before analysis. Chromatography was performed using an Agilent 1290 Infinity UPLC. Ten microliters of each sample were injected onto a ZIC-pHILIC column (EMD Millipore, Billerica, MA) with dimensions of 150 × 4.6 mm, 5 μm. Metabolites were separated using an acetonitrile/H_2_O with 20 mM ammonium carbonate (pH 9.2) gradient over a 29-min period. A 10-min re-equilibration time was carried out in between injections. Detection was performed using an Agilent 6550 quadrupole-time-of-flight (QToF) mass spectrometer, operated in both negative and positive modes. Full scan MS data was collected from m/z 70–1000 and metabolites were identified in an untargeted manner by looking within 10 ppm of the expected m/z values. Real-time mass calibration was performed throughout the duration of sample analysis. Data was processed using a publically available software package, MAVEN^19^. Area under the chromatographic peak for each metabolite was calculated and exported to assess differences in metabolite abundances.

#### Targeted ^13^C metabolic tracer analysis

[U-^13^C]glutamate (5 mM) or [U-^13^C]malate (2.5 mM) was added to the mitochondrial suspensions, as indicated in the text. Metabolites extraction was performed similar to the untargeted metabolite analysis, with the exception that in the supernatants, 1 mM methionine was added for normalizing volumes, as it also yields a highly recognizable signature during the analysis. Dried lysates were derivatized using a two-step protocol. Samples were first treated with 2% methoxamine in pyridine (40 μL, 1 h at 60 °C), followed by addition of N-(tert-butyldimethylsilyl)-N-methyl-trifluoroacetamide, with 1% tert-butyldimethylchlorosilan (50 μL, 1 h at 60 °C). Samples were transferred to glass vials for GC–MS analysis using an Agilent 8890 GC and 5977B MSD system. 1 μL of sample was injected in splitless mode with helium carrier gas at a rate of 1.0 mL^.^min^−1^. Initial GC oven temperature was held at 100 °C for 1 min before ramping to 160 °C at a rate of 10 °C^.^min^−1^, followed by a ramp to 200 °C at a rate of 5 °C^.^min^−1^ and a final ramp to 320 °C at a rate of 10 °C^.^min^−1^ with a 5 min hold. Compound detection was carried out in scan mode. Total ion counts of each metabolite were normalized to the internal standard norleucine (supernatants) or methionine (pellets).

#### Quinones extraction

2 mg mitochondria were suspended in a solution of 5 mM ferricyanide, 100 mM Tris, pH 8.0. Proteins were precipitated by addition of methanol triple the aqueous volume, followed by extraction three times with light petroleum (bp. 40–60 °C). After evaporation of the solvent from the extract, the residues were dissolved in ethanol and the absorption measured at 275 nm before and after stepwise additions of a 5 g/L borohydride solution in a 1 cm path length cuvette. Quinone concentration was calculated with the oxidized-reduced difference absorbance Δε_275 nm_ = 12.5 mM^−1^ cm^−1^, reported in^20^.

#### Blue-native gel electrophoresis, western blot and immunodetection

The analysis of the super-assembly state of Complex I was performed as described previously^21^. Briefly, frozen mitochondrial pellets were resuspended at a protein concentration of 10 mg/ml in solubilization buffer (1.5 M aminocaproic acid, 50 mM Bis–Tris/HCl pH 7.0) and the mitochondrial membranes were solubilized using a digitonin to protein ratio of 4:1. The cleared lysate was mixed with sample buffer [0.75 M aminocaproic acid, 50 mM Bis–Tris–HCl pH 7.0, 0.5 mM EDTA, 5% SERVA Blue G (Coomassie Brilliant Blue G-250)] and approximately 15 µg of mitochondrial protein were loaded in each lane of precast native 3%-13% gradient gels (Invitrogen) and run according to the manufacturer’s instructions. Once the front reached the end of the gel, the electrophoresis was stopped and the native complexes were blotted onto PVDF membranes and immunodetected. Complex I was specifically detected using an antibody against NDUFS1 (RRID: AB_2687932) and Complex II was detected with an anti-SDHB antibody (RRID: AB_301432). For signal detection, Goat anti-Rabbit IgG (H + L) Highly Cross-Adsorbed Secondary Antibody, Alexa Fluor™ Plus 800 (RRID: AB_2633284) and Goat anti-Mouse IgG (H + L) Cross-Adsorbed Secondary Antibody, Alexa Fluor™ 680 (RRID: AB_2535723) were used and the images were captured using an Odyssey CLx instrument (Li-Cor Biosciences).

#### Reagents

Mitochondrial substrates were dissolved in bi-distilled water and titrated to pH 7.0 with KOH. ADP was purchased as a K^+^ salt of the highest purity available (Merck) and titrated to pH 6.9. Concentrations of glutamate (G), malate (M), succinate (S), fumarate (Fum) and oxoglutarate (Og) were always 5 mM when present, unless otherwise indicated. ADP concentrations were 2 mM. Rotenone (Rot, 1 µM), myxothiazol (Myx, 0.1 µM), stigmatellin (Stigm, 0.5 µM) carboxyatractyloside (CAT, 1 µM), SF6847 (SF, 0.25 µM), piericidin A (Pierc, 1 µM), pyridaben (Prdb, 1 µM), atpenin A5 (Atpn, 1 µM), malonate (Mna, 5 mM), cyanide (NaCN or KCN, CN, 1 mM), arsenite (Arsn, 1 mM or 2 mM). Both myxothiazol and stigmatellin block CIII; they have been used in our experiments according to availability.

### Quantification and statistical analysis

#### Statistics

Data are presented as averages ± SEM. Significant differences between two groups were evaluated by Student's t-test or Mann–Whitney U Test if normality failed. Significant differences between three or more groups were evaluated by one-way ANOVA or ANOVA on Ranks if normality failed; statistical significance was accepted when *p* < 0.05 (shown as *). The number of experiments is indicated in the figure legends. Regarding figure panels 4K, 4L, 4M and supplementary figure panels 14E and 14F: Combined standard uncertainty was calculated using Gauss’ law of error propagation with linear approximation using the NIST uncertainty machine v1.5. Subsequently, covariance between succinate isotopologue amount fractions and between 2-oxoglutarate isotopologue amount fractions were taken into account, although the correction was negligible. No other consistent correlation was found among the uncertainties.

## Results

### Complex I remains partially active during acute anoxia

**Figure 1:**
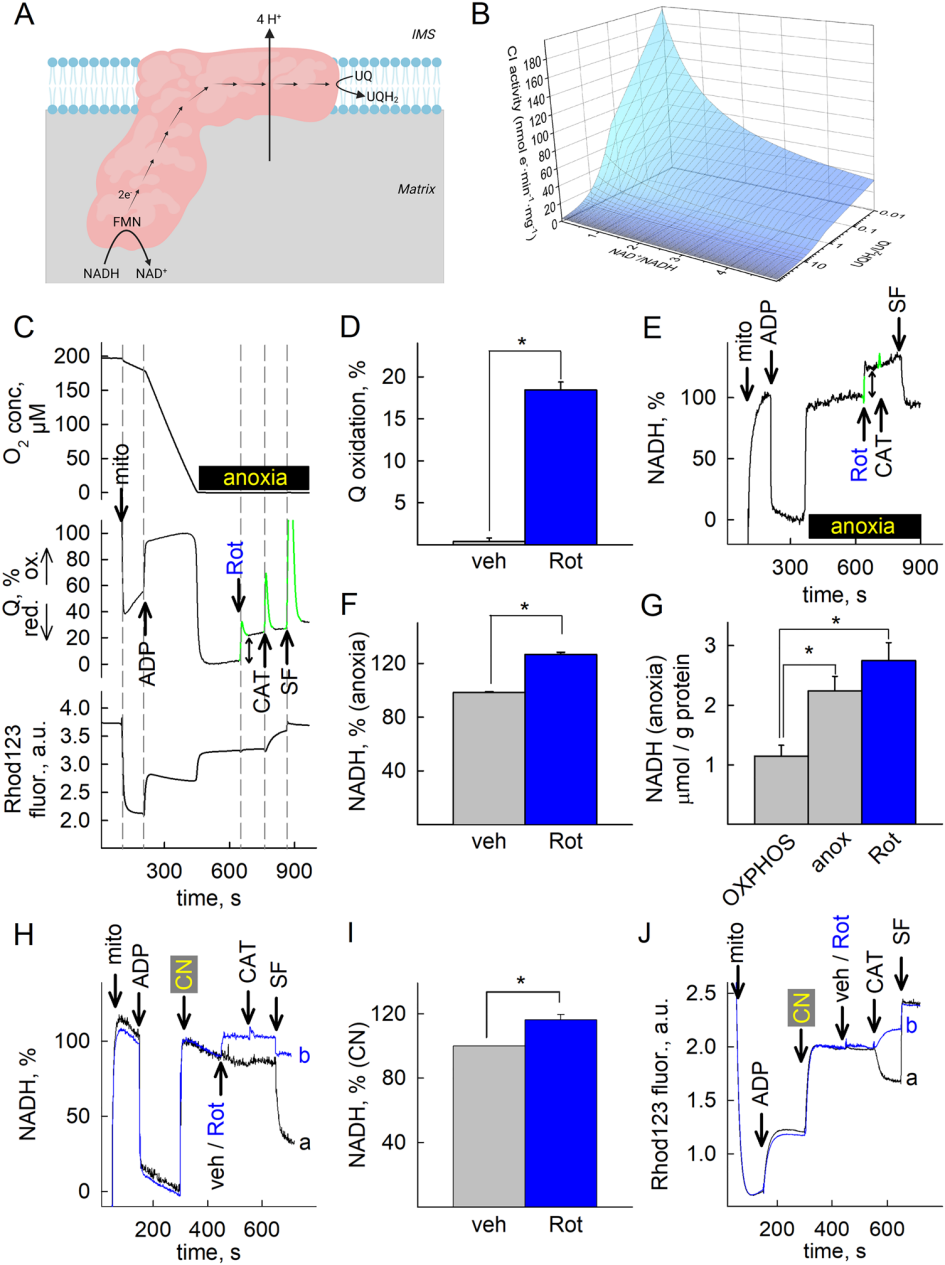
Complex I remains partially active during acute anoxia. (**A**) Scheme illustrating the reaction catalyzed by CI (created with BioRender.com). (**B**) 3D plot depicting CI activity as a function of UQH_2_/UQ, NAD^+^/NADH for Δ*Ψ*_mt_ −100 mV, pH_in_ 7.35 and ΔpH  0.1; these values were taken from measurements in the present study and from those published in^106^. CI activity (J_CI) is the product of the rate equation formulated as J_CI = Vmax × [NADH]/[NAD^+^]total pool × [Q]/[Q]total pool × F_T_, where F_T_ is thermodynamic drive. All concentrations are in matrix, [NAD]total pool = [NAD^+^] + [NADH]; ETS-reactive [Q]total pool = [UQ] + [UQH_2_]. (**C**) Oxygen concentration (top panels, in μM) recorded simultaneously with UQ redox state (middle panels) and rhodamine 123 fluorescence (Rhod123, bottom panels) in isolated mouse liver mitochondria; panels are aligned in the x-axis. (**E**) NADH autofluorescence in isolated mouse liver mitochondria undergoing anoxia. Mitochondria (mito), ADP (2 mM), rotenone (Rot, 1 μM), carboxyatractyloside (CAT, 1 μM), SF (SF6847, 0.25 μM) were added where indicated. Substrates were glutamate and malate (5 mM each) present in the buffer prior to addition of mitochondria. (**D,F**) Quantification of the vehicle (veh) or rotenone-induced changes in Q (**D**) or NADH (**F**) signal in anoxic mitochondria, as illustrated by the bidirectional arrows in (**C**) (middle panel) and (**E**), respectively. The peaks colored in green in panels C and E are artefactual caused by the additions of the drugs and are removed from all further quantifications. * *p* < 0.05. (**G**) The amount of total NADH measured in the alkaline extract (in μmol/g of protein) at OXPHOS (grey) (n = 3), anoxia (grey, anox) (n = 4) and rotenone added in anoxia (blue, Rot) (n = 4) from isolated mitochondria; *p < 0.05. (**H**) The effect of rotenone (blue, b trace) added to mitochondria in which chemical anoxia was induced by 1 mM KCN (indicated by CN in the panel) on isolated mouse liver mitochondria compared to veh (black, a trace). (**I**) Quantification of the vehicle or rotenone induced changes in NADH signal in CN-treated mitochondria (n = 4, * p <0.05). (**J**) Rhodamine 123 fluorescence traces of the experiments performed exactly as in (**H**).

Mindful that CI oxidizes one molecule of NADH to NAD^+^ by reducing UQ to UQH_2_ in an equimolar manner while pumping four protons out of the matrix as shown in Fig. 1A, Jin and Bethke derived a model for CI activity using non-equilibrium thermodynamics^22^. We used their rate equation to generate a 3D plot of CI activity (expressed in nmol e^−^min^−1^ mg^−1^) and input very wide ranges of UQH_2_/UQ and NAD^+^/NADH ratio reported in the literature^23–27^. As shown in Fig. 1B, when UQH_2_/UQ is > 10 and NAD^+^/NADH < 1 mimicking anoxic conditions, CI activity is predicted to retain < 20% of its theoretical maximum. We therefore set to investigate (i) if CI activity can indeed be experimentally demonstrated under anoxic conditions and (ii) whether the fluxes of the reaction products, i.e. NAD^+^ and UQH_2_ are sufficiently high for maintaining downstream processes and—if yes—to which extent. As a first step, we set up an assay to simultaneously monitor the ETS-reactive Q redox state, NADH autofluorescence, O_2_ concentration and membrane potential (Δ*Ψ*_mt_) during anoxia, in a (sub)second scale. We mostly used isolated mouse liver mitochondria because of the high yield, purity and level of intactness achieved in this preparation^28^, however, several experiments were performed and interpretations verified by using brain and heart mitochondria (see below and supplementary Figs. 4 and 5). [O_2_] was detected polarographically, Δ*Ψ*_mt_ fluorimetrically (inferred from rhodamine 123 preloading), NADH by its autofluorescence, and the ETS-reactive Q redox state electrochemically using coenzyme Q_2_ as a mediator between the mitochondrial UQ pool and the electrodes ^14,17^. All species that have a connection to endogenous ubiquinone through the so-called “coenzyme Q junction” ^29–31^ can contribute to the electrochemical reaction, including the endogenous ubiquinone itself present in the mitochondrial inner membrane, by reducing Q_2_. The electrochemical properties of the two (endogenous and Q_2_) are virtually identical owing to the difference being only in the distant hydrophobic chain and therefore can be considered to be close to an equilibrium. However, it is still possible to observe different properties due to the differential solubility and enzyme affinities. Other artificial reactions may also be considered, such as oxidation by dissolved oxygen, but over the experiments conducted, qualitatively no significant deviation was observed compared to just treating it as a representation of the endogenous ETS-reactive ubiquinone (Q) pool, which it is in equilibrium with. This is further supported by the lack of other electrochemically active compounds during cyclic voltammetry shown in supplementary Fig. 17. Rhodamine 123 fluorescence indicative of Δ*Ψ*_mt_ (arbitrary units, a.u.) was recorded separately from NADH autofluorescence due to spectral overlap. As shown in Fig. 1C [O_2_], UQ % and rhodamine 123 fluorescence (indicative of Δ*Ψ*_mt_) were measured simultaneously in the same sample (panels are aligned in the x-axis); in Fig. 1E NADH % is depicted, measured in another sample simultaneously to [O_2_] and Δ*Ψ*_mt_. Mitochondria were added where indicated, followed by ADP inducing OXPHOS respiration. The CI-specific inhibitor rotenone (Rot) or vehicle (veh) was added before (supplementary Figs. 1A, D) or after (Fig. 1C, E, and supplementary Figs. 1B, E) the chamber was depleted from O_2_ (in the figure panels marked as “anoxia”). Upon induction of anoxia the oxygen sensor detects no further changes in O_2_ concentration (Fig. 1C and supplementary Fig. 1B, top panels); for detailed description of the sensor response during the transition from normoxic to anoxic conditions, see ^32^. Anoxia was associated with near simultaneous, precipitous reduction of UQ (Fig. 1C, supplementary Fig. 1B, middle panels) and increases in rhodamine 123 fluorescence (indicative of depolarization, Fig. 1C and supplementary Fig. 1B bottom panels) and abrupt increases in NADH autofluorescence (Fig. 1E and supplementary Fig. 1E). As expected, ADP and anoxia conferred an increase- *vs* a decrease in Q oxidation levels (Figs. 1C and supplementary Fig. 1B, middle panels), respectively, and accordingly, a decrease- *vs* an increase in NADH levels (Figs. 1E and supplementary Fig. 1E). However, it is also obvious that rotenone inhibited UQ reduction to UQH_2_ whether O_2_ was present (supplementary Fig. 1A, middle panel) or not (Fig. 1C, middle panel). The spikes (pseudocolored in green in middle panels 1C, supplementary Fig. 1B) are artefacts produced by the addition of chemicals; from such experiments we pooled the values in the Q signals conferred by the additions (as indicated by the bidirectional arrow in 1C, middle panel) and show them in bar graphs in figure panel 1D. Furthermore, addition of rotenone to anoxic mitochondria yielded a further increase in NADH fluorescence (Fig. 1E). Pooled values in NADH signals conferred by the additions (indicated by the bidirectional arrow) are shown in bar graphs in figure panel 1F (NADH levels after the addition of ADP and commencement of anoxia were arbitrarily assigned as the 0% and 100% internal reference points of the scale, respectively). The rotenone-induced changes in NADH could also be observed when mitochondria were treated with NaCN (1 mM) to confer ‘chemical anoxia’, instead of true anoxia. Representative traces are shown in Fig. 1H; in trace a, vehicle (veh) is added where indicated, and in trace (b), rotenone. Quantification of the rotenone-induced change in NADH fluorescence in CN-treated mitochondria is shown in Fig. 1I. Accordingly, changes in rhodamine 123 fluorescence are similar if mitochondria are rendered chemically anoxic with CN *in lieu* of absence of oxygen, shown in Fig. 1J. Notably, rotenone conferred a CAT-induced depolarization when added after CN (compare trace b, with trace a, in Fig. 1J); as it will be shown later on, this reflects that NAD^+^ provision to OgDHC by residual CI activity is critical for maintenance of ANT operation in ‘forward’ mode^12^. The effect of CN (or azide) on Q redox state cannot be investigated with the Q-sensor because of direct interference with the electrode^17^. The rotenone-induced increases in reduced Q and NADH suggest that CI was operating in forward mode; although CI is fully reversible ^33,34^ the conditions allowing reversibility *in organello* are extreme: by plotting CI forward- vs reverse operation as a function of UQH_2_/UQ, NAD^+^/NADH, matrix pH (pH_in_) and ΔpH across the inner mitochondrial membrane and assuming that anoxia clamps Δ*Ψ*_mt_ of isolated mouse liver mitochondria to ~ − 100 mV^35^, there can be only two circumstances upon which CI may operate in reverse: as shown in supplementary Fig. 6, CI operates in reverse if either (i) UQH_2_/UQ > 100 an NAD^+^/NADH = 10 and pH_in_  7.35 and ΔpH is between 0.8 and 1.0 (supplementary Fig. 6D), or if (ii) UQH_2_/UQ > 100 and NAD^+^/NADH = 10 and pH_in_ 8.35 and ΔpH is between 0.5 and 0.8 (supplementary Fig. 6E). Alternatively, these values could reach more realistic numbers if mitochondria became more polarized during anoxia (delineated by the dotted areas in the same panels), which on the other hand, is extremely unlikely. Overall, it is impossible to achieve such conditions in intact mitochondria. Finally, as shown in supplementary Fig. 1A and supplementary Fig. 3A (bottom panels) inhibition of the adenine nucleotide translocase (ANT) by carboxyatractyloside (CAT) in rotenone-inhibited, but not anoxic mitochondria leads to a gain in Δ*Ψ*_mt_, commensurate with our previous data showing that ANT remains in forward mode when CI is inhibited^12^. Likewise, addition of CAT to anoxic mitochondria (in the absence of rotenone, supplementary Figs. 1B and 3B, bottom panels) also leads to a gain in Δ*Ψ*_mt_, commensurate to our data published before^35^. However, CAT led to a loss of Δ*Ψ*_mt_ when CI was inhibited in anoxic mitochondria (Fig. 1C and supplementary Fig. 3C, bottom panels), implying ANT reversal^36^. The observation that the ANT was operating in forward mode when oxygen was absent (or CIV was inhibited by CN) or CI was inhibited, and in reverse mode when both oxygen was absent and rotenone was added (see also Fig. 1J regarding chemical anoxia) hints that rotenone affects mtSLP, the primary determinant of ANT directionality when OXPHOS is inhibited^37^. Alternatively, rotenone could have an off-target effect anywhere on pathways converging towards mtSLP, dictating ANT directionality. However, the alternative CI inhibitors pyridaben (Prdb) and piericidin A (Pierc) reproduced the effect of rotenone on Q redox state during anoxia (see representative traces in supplementary Fig. 3D, E, middle panels; mean signals are shown in supplementary Fig. 1C). Accordingly, a further increase in NADH autofluorescence was observed when CI inhibitors were added during anoxia (supplementary Fig. 3F, G, middle panels; mean signals shown in supplementary Fig. 1F). It is noteworthy that the effect of the inhibitors on the Q signal was unequal (strength of effect, in descending order: rotenone > pyridaben > piericidin A), while all three exerted the same effect in increasing NADH autofluorescence when added on top of anoxia. This probably reflects that although rotenone, pyridaben and piericidin A share a common binding domain at- or in the vicinity of the UQ reduction site^38^, the exact sites of action can be more than one in addition to being partially overlapping^39^. Nonetheless, addition of Prdb or Pierc during anoxia led to a CAT-induced loss of Δ*Ψ*_mt_, implying ANT reversal and therefore absence of mtSLP (supplementary Fig. 3D–G lower panels). As expected, the Complex II inhibitor atpenin A5 (Atpn) also led to a CAT-induced loss of Δ*Ψ*_mt_, implying ANT reversal and therefore absence of mtSLP (supplementary Fig. 3H). The lack of effect on these (and all other inhibitors used in this study) on inducing permeability transition during anoxia (detected by changes in light scatter of the organelles) is shown in supplementary Fig. 7A. The potential connection of CI activity to mtSLP is provision of NAD^+^ for OgDHC which yields succinyl-CoA, in turn supporting mtSLP^40^. Because the rotenone-induced changes in NADH fluorescence and Q redox state in anoxia were smaller than those conferred in the presence of oxygen (compare Fig. 1E with supplementary Fig. 1D for NADH and Fig. 1C with supplementary Fig. 1A, middle panels for UQ), we deduced that CI activity is only partially active during anoxia. To further support our claim, we measured the total amount of NADH that could be extracted from mitochondria during OXPHOS respiration, after anoxia, and after addition of rotenone in anoxia. As shown in figure panel 1G, anoxia led to an increase in the total amount of NADH compared to OXPHOS (grey bar); further addition of rotenone (blue bar, Rot) led to a further increase in NADH, which is small compared to the anoxic state, mindful that the rotenone-induced increase in NADH fluorescence in anoxic, intact mitochondria is fairly small (see Fig. 1 panel E), in addition to the notion that total amount of NADH is subject not only to oxidation mediated by CI, but other matrix entities as well, see supplementary Table 1.

To address the possibility that the Oroboros chamber and/or the 3D-printed plug (used for experiments shown in supplementary Fig. 7 with standard polymethacrylate cuvettes) where true anoxia was implied but not actually achieved due to potential leakage of gases through the pins through which the Q electrode and chemicals are added, we manufactured air-tight borosilicate glass chambers. These chambers consist of a chamber and a glass lid and in-between a PTFE/Silicone septum (Supelco, 27095-U) is in place that exhibits severely limited oxygen permeability (appreciable diffusion of oxygen and other gases through these septa occurs within weeks, much longer than the time frame of our experiments that lasted 10–20 min). This septum can be pierced by Hamilton syringes and self-seals immediately upon retrieval of the syringe. Piercing and self-sealing can be reliably applied at least four times per septum. Schematics and pictures of such a chamber are shown in supplementary Fig. 2A–G. All chemicals injected through the septum are gassed with Argon 5.0 (99,999% pure). Borosilicate glass of the chamber prevents near-UV light (required for NADH fluorescence measurements) only to a minor extent; NADH spectra for a polymethacrylate cuvette (suitable for UV) and for the glass chamber are shown in supplementary Fig. 2H and supplementary Fig. 2I, respectively. A far-left shoulder of NADH excitation (black trace, supplementary Fig. 2H, around 260 nm) is eliminated when the NADH spectrum is performed in the glass chamber (supplementary Fig. 2I), but that did not affect our measurements because excitation was set to 340 ± 5 nm where less than 10% loss was observed in the exc/em peaks. Rhodamine 123 fluorescence was completely unaffected; as shown in supplementary Fig. 2J, rhodamine 123 fluorescence exhibited an abrupt splay towards higher values (implying depolarization), which is expected upon commencement of anoxia. Subsequent addition of CAT in the presence (blue trace, b) but not in the absence (black trace, a) of rotenone led to a further depolarization, exactly in line with the notion that ANT remains in forward mode when CI is not pharmacologically inhibited^12^. Likewise, addition of rotenone in anoxic mitochondria led to an increase in NADH fluorescence (supplementary Fig. 2L) compare to vehicle (supplementary Fig. 2K). Since the traces obtained from mitochondria suspended in the air-tight glass chamber (rhodamine fluorescence, NADH fluorescence and response to all inhibitors and treatments) were almost identical to those obtained with the Oroboros chamber, we were confident that no minor oxygen leakage was occurring in the latter instrument, where Q redox state can be also addressed; thus, our interpretations were not confounded from a potential oxygen leakage. Accordingly, the presence of CIII inhibitor myxothiazol did not prevent the rotenone-induced increase in NADH fluorescence (supplementary Fig. 2M), but addition of 5 mM succinate (S) prior to rotenone completely prevented this effect (supplementary Fig. 2N). This hinted to the potential role of Complex II operating in reverse (and thus prevented from doing so by excess succinate), providing UQ to a residually-operating CI, as suggested in^41^. Quantification of technical replicates is shown in a bar graph in supplementary Fig. 2O, P. Fumarate is a very poor substrate (largely due to slow uptake) in isolated mitochondria; experiments where the ratio of succinate/fumarate was experimentally altered using permeabilized mitochondria is addressed below (see supplementary Fig. 10). The effects of rotenone conferring a CAT-induced depolarization in anoxic mitochondria concomitant to a rotenone-induced increase in NADH fluorescence was also observed in intact mitochondria obtained from brains (supplementary Fig. 4) and hearts of mice (supplementary Fig. 5).

### Endogenous Q pool is rapidly declining, supporting a brief, partial CI activity during acute anoxia

NADH availability could not have been a factor of finiteness as it is in excess during the anoxic insult. On the other hand, mindful that the amount of CI in rodent liver is ~ 6 pmol/mg protein^42^, the total CoQ content is higher than 3 nmol/mg^43,44^, the amount of CoQ bound to mitochondrial proteins is between 10 and 32%^45^, CoQ binds stoichiometrically with each site in the ETS complexes^46–48^, and the catalytic turnover of CI is 10^4^/min^42,49^ it should take only a few seconds for Complex I to reduce all available CoQ. To address the ETS-reactive pool of Q supporting CI activity during anoxia we recorded the % change in the Q signal as a function of time elapsed from commencement of anoxia until the addition of CI inhibitors. As shown in supplementary Fig. 9, rotenone (A–C) or pyridaben (D) or piericidin A (E) was the CI inhibitor when using either glutamate (G) and malate (M), or oxoglutarate (Og), or oxoglutarate and malate as fueling substrates. Representative experiments used to estimate Q % redox states as a function of CI inhibitor and/or substrate(s) are shown in supplementary Fig. 8 panels A-G, middle panels. It is evident that the longer the time elapsed from commencement of anoxia until addition of CI inhibitors the smaller the relative change of the Q signal. During anoxia the ability of permeabilized mitochondria (using alamethicin) to oxidize exogenous NADH in a rotenone-sensitive manner is decreasing in a time-dependent manner, see supplementary Fig. 9F, G. As expected, CAT induced loss of Δ*Ψ*_mt_ in all conditions irrespective of inhibitor or substrate(s) present or time elapsed, implying ANT reversal (supplementary Fig. 8A–G, bottom panels). The discrepancy between the fact that the amount of reducible CoQ is sufficient for only a few seconds with the extended time frames observed in our experiments shown in supplementary Figs. 8 and 9 cannot be reconciliated.

To address this further, we fed mice with a diet devoid of vitamin K_3_ over the course of approximately 3 weeks. Omission of vitamin K_3_/K_1_ from the diet has been reported to influence the mitochondrial Q/menaquinones pool in laboratory rodents respectively^50,51^. After each week, prothrombin time (PT) was measured as an indicator of vitamin K status^52–54^. As shown in supplementary Fig. 8H, PT was increased in mice kept in vitamin K_3_-deficient diet. In post-day 16 after implementing the vitamin K_3_-deficient diet total liver Q levels were diminished, see supplementary Fig. 8I; however, by post-day 24 the trend was reversed, which means the dietary intervention did not affect CoQ content. The Q redox state upon addition of rotenone did not differ in anoxic mitochondria obtained from mice kept on vitamin K_3_-deficient diet for 3 weeks *vs* control littermates (supplementary Fig. 8J). Although it is possible to use transgenic mice with constitutive ablations in genes coding for proteins participating in the ubiquinone synthesis pathway, such mitochondria would inherently exhibit severe OXPHOS deficiencies to the point that ANT directionality could not be addressed, since such assays require fully polarized mitochondria with very high respiratory control ratios^37^. Despite that we could not manipulate the pool of endogenous CoQ by dietary means, the data above support the notion that UQ availability is critical for CI activity during anoxia only for a few seconds. However, this does not exclude the possibility of UQH_2_ also being simultaneously oxidized by some other entity during anoxia. We have also addressed the possibility that the mitochondrial transhydrogenase (encoded by NNT) may have some contribution to NADH/NAD^+^ ratio and as an extension of this to UQ/UQH_2_; for this, we compared liver mitochondria isolated from C57Bl/6N mice vs those obtained from the 6J sub-strain; mice of the latter strain do not exhibit the transhydrogenase^55^. The latter effect may bear relevance to our study because in light of a disability to form respiratory complexes, sources of electron for the ETS may come from elsewhere. However, as shown in supplementary figure panels 8K and 8L for Q oxidation (%) and NADH %, respectively, there were no statistically significant differences between the results obtained from rotenone added to anoxic mitochondria isolated from the livers of 6N (smooth) vs 6J (cross-hatched) mice. Thus, the only potential source of UQ to CI could be from CII reversal, see below.

### Fumarate reductase activity in permeabilized mitochondria

**Figure 2:**
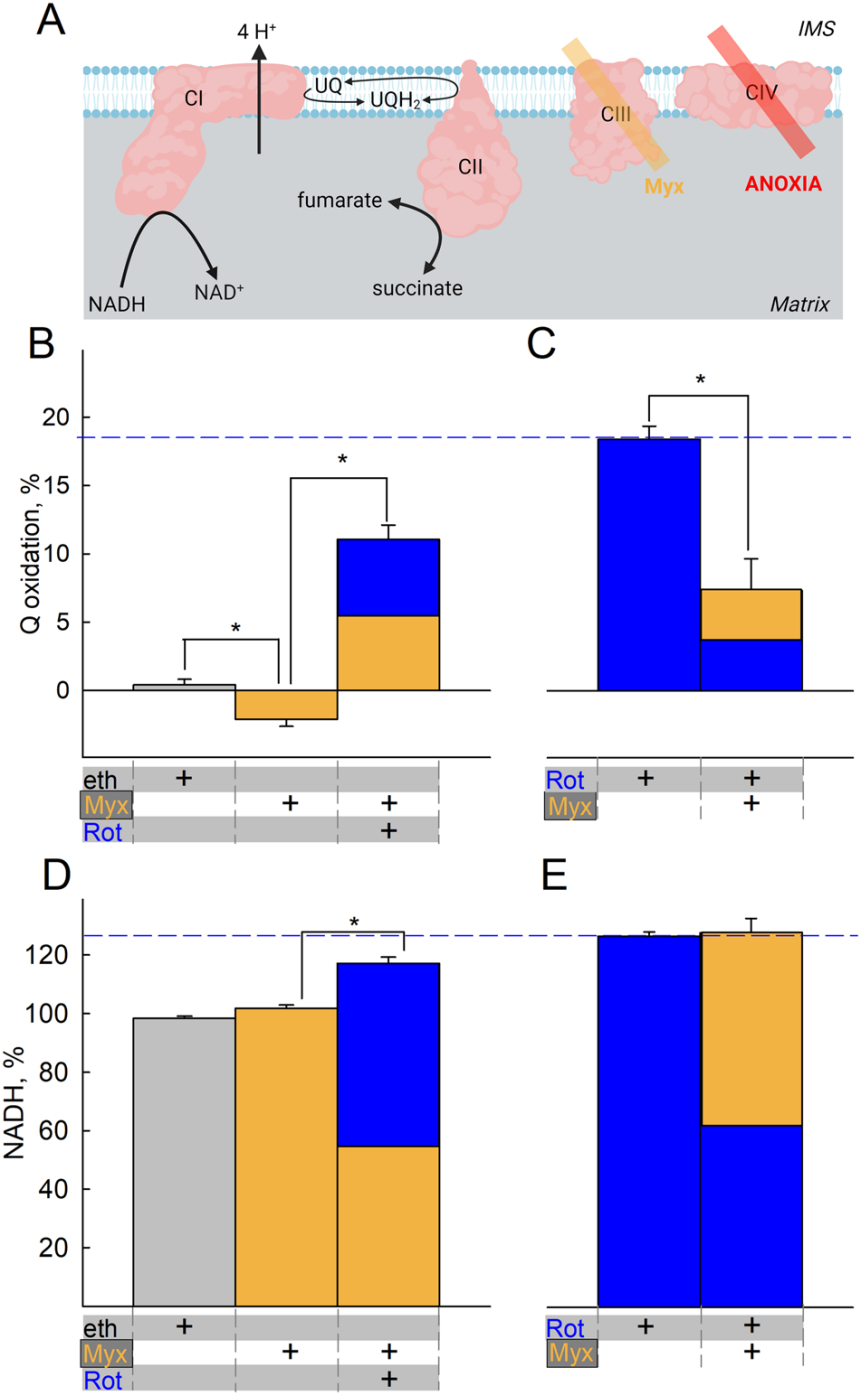
CIII does not mask CI operation during anoxia. (**A**) Scheme illustrating the action of inhibitor(s) used downstream of CI in the experiments shown in the figure (created with BioRender.com). Quantification of inhibitor-induced changes in UQ (**B,C**) or NADH (**D,E**) signal. Vehicle or inhibitors present as indicated by the plus ( +) signs at the bottom of the panels. Substrates were glutamate and malate (5 mM each) present in the buffer prior to addition of mitochondria. *Eth* ethanol (grey), *Myx* myxothiazol, 0.1 μM (orange), *Rot* rotenone, 1 μM (blue). The additions of the compounds mentioned in the bottom of each panel were made from top to bottom. Data pooled from 3 to 37 independent experiments. *p < 0.05.

The concept of combined Complex I activity in forward mode and Complex II in the reverse collectively referred to as “NADH:fumarate reductase activity” originates from Sanadi and Fluharty^56^. However, mindful that this activity is ~ 100 times smaller than the physiological NADH:Q10 reaction of Complex I^49^, we set to estimate the activity of CII reversal in permeabilized mitochondria, as this is imperative for the overall NADH:fumarate reductase activity to manifest. The experimental protocol was to evaluate the rate of rotenone-sensitive NADH oxidation activity in permeabilized mitochondria that were rendered anoxic, and variate the fumarate/succinate (Fum/Succ) ratio. Anoxia was applied as either ‘true’ by allowing mitochondria to run out of oxygen in the air-tight glass chambers or ‘chemical’ by inclusion of KCN. Permeabilization was achieved by adding the pore-forming peptide alamethicin to the media. Representative traces for KCN-induced anoxia and NADH oxidation rate is shown in supplementary Fig. 10A, and for true anoxia in supplementary Fig. 10C. As shown in supplementary Fig. 10B and 10D for KCN and true anoxia respectively, we were unable to demonstrate a statistically significant tendency in which greater concentrations of fumarate ‘pushing’ CII towards higher reversal rates would yield greater rates of NADH oxidation by CI (by means of providing more UQ). This is not surprising, considering the very low rate at which CII reverses. Indeed, as Spinelli has shown^41^, CII reversal by addition of fumarate to respiration-impaired mitochondria can only be observed upon very long incubation periods (see Fig. 2 panel I on the right in reference^41^). There, it was necessary to wait several hours, until the rate of succinate produced by CII reversal could be reliably detected. However, it is not necessary to have a high rate of CII reversal in order for CI to provide a sufficient amount of NAD^+^ for OgDHC; it is emphasized that the main point of the present work is to demonstrate provision of ATP from succinate-CoA ligase for maintaining ANT in forward mode, during anoxia; that requires succinyl-CoA which comes from OgDHC. These are addressed in subsequent sections (Figs. 4 and 5), but prior to that we investigated the potential contribution of CIII as a means of UQH_2_ oxidizer, when CIV is not operational.

### CIII does not affect CI operation during anoxia to an appreciable extent

Having established that CI exhibits residual activity even in anoxia (true, or chemical) thus reducing UQ to UQH_2_ and mindful that the latter is the substrate of CIII, we tested the effect of CIII inhibitor myxothiazol (Myx, Fig. 2A) on Q- and NADH/NAD^+^ redox levels. As shown in Fig. 2 panel B, the presence of myxothiazol led to a minor though statistically significant decrease in UQH_2_ oxidation levels when added in anoxia, reflecting a minor CIII activity. This change was enhanced by addition of rotenone prior to myxothiazol (both added after anoxia, Fig. 2C), but UQH_2_ levels were higher implying that in anoxia the changes in Q pool are mostly dictated by CI and not CIII. The effect of rotenone when added after myxothiazol still implied residual CI activity even with CIII already inhibited; the effects of the alternative CI inhibitors pyridaben and piericidin A are shown in supplementary Fig. 11A. Similarly, the presence of myxothiazol before (Fig. 2D) or after (Fig. 2E) rotenone (and supplementary Fig. 11B for pyridaben and piericidin A) did not abolish NADH changes conferred by the CI inhibitors. These results argue that in anoxia there is a minor CIII activity, but most importantly CI residual activity is not solely influenced by inhibition of CIII. The electron acceptor(s) for this minor CIII activity were not investigated further.

### CII operates amphidirectionally, reducing fumarate during acute anoxia

**Figure 3:**
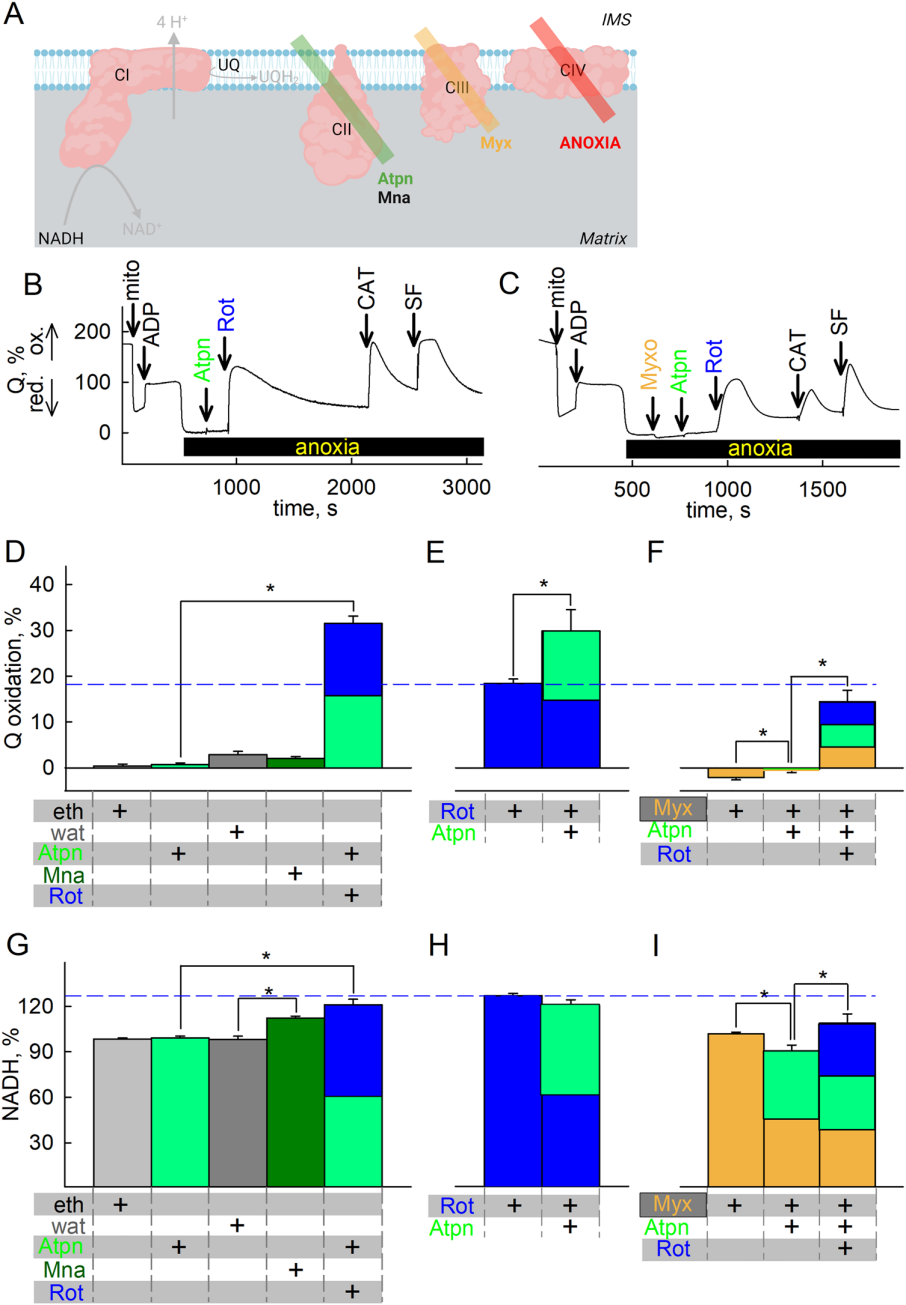
CII operation during acute anoxia in relation to Q redox state. (**A**) Scheme illustrating the action of inhibitor(s) used downstream of CI in the experiments shown in the figure (created with BioRender.com). (**B,C**) Representative traces of inhibitor-induced changes in Q redox state; quantification of inhibitor-induced changes in Q redox state (**D–F**) or NADH (**G–I**) signal. *Eth (grey)* ethanol; *wat (dark grey)* water; *Mna, 5 mM (dark green)* malonate; *Atpn, 1 μM (green)* atpenin A5; *Myx* myxothiazol, 0.1 μM (orange); *Rot* rotenone, 1 μM (blue). In (**D–I**) the additions of the compounds mentioned in the bottom of each panel were made from top to bottom. Data pooled from 3 to 37 independent experiments. *p < 0.05.

**Figure 4:**
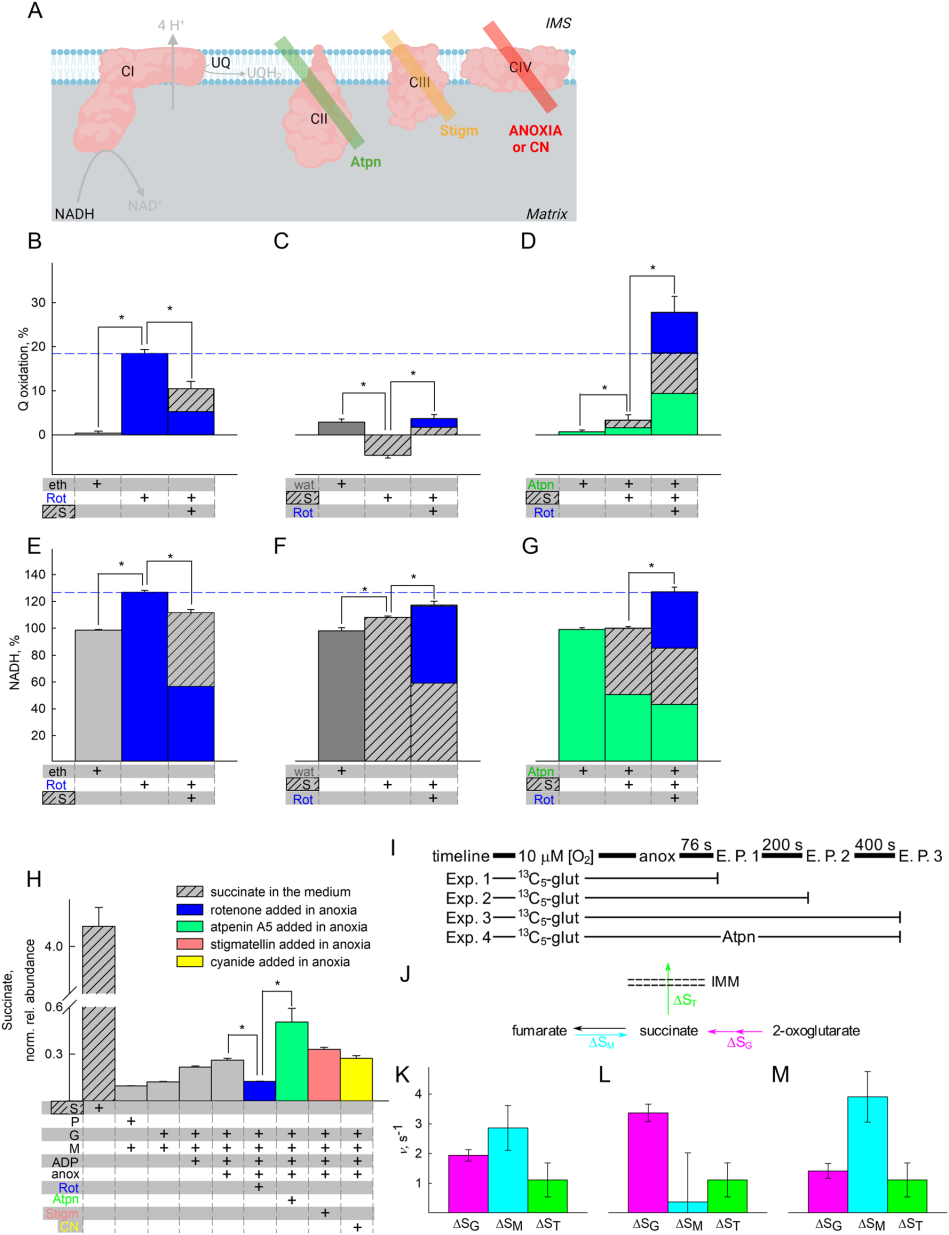
Amphidirectional CII operation during acute anoxia. (**A**) Scheme illustrating the action of inhibitor(s) used downstream of CI in the experiments shown in the figure (created with BioRender.com). Quantification of rotenone-induced changes in Q redox state (**B–D**) or NADH (**E–G**) signal. *Eth (grey)* ethanol, *Rot* rotenone, 1 μM (blue); *S* succinate, 5 mM (striped grey). (**H**) Untargeted metabolite analysis of succinate (S) present in the pellets of mitochondria treated with the conditions indicated in the panel. The y-axis illustrates normalized, log transformed, and scaled peak area. The additions of the compounds mentioned in the bottom of each panel were made from top to bottom. *S* succinate, 5 mM (striped grey); *G* glutamate, 5 mM; *M* malate, 5 mM; *P* pyruvate, 5 mM; *Rot (blue)* rotenone, 1 μM; *Atpn (green)* atpenin A5, 1 μM; *Stigm (orange)* 0.5 μM: stigmatellin; *CN (yellow)* cyanide, 1 mM. Data pooled from 3 to 37 independent experiments. * *p* < 0.05. (**I**) A cartoon representing the experimental timeline and extraction points (E.P.). Experiments always started the same as previously described, except 2.5 mM unlabeled glutamate was present and 2.5 mM [U-^13^C]-labelled glutamate was added just before anoxia when [O_2_] was ~ 10 μM. Just prior to extraction, 500 μM N-ethylmaleimide, 1 μM rotenone, 1 μM atpenin, 1 μM myxothiazol, 1 mM arsenite, 100 μM *p*-hydroxymercurybenzoate, 100 μM aminooxyacetate and 1 mM cyanide were added to the suspension in the closed chamber. Samples were extracted as described in the methods section. (**J**) A model for pathways leading to- and from succinate. Note the directionality of the arrows: ΔS_M_ is positive in the fumarate to succinate direction and ΔS_T_ is positive in the export direction. Because no succinate was added externally, it is reasonable to assume that succinate exits through the IMM. (**K–M**) Average rates of succinate formation and transport expressed as velocity (*ν*). Rates are represented instead of absolute amounts to make different timeframes comparable: (**K**) 0–600 s; (**L**) 0–200 s; (**M**) 200–600 s. Magenta signifies succinate from glutamate, cyan from malate, green from transport across IMM. For (**K–M**) Combined standard uncertainty was calculated using the Gauss’ law of error propagation using linear approximation using the NIST uncertainty machine v1.5. Subsequently, covariance between succinate isotopologue amount fractions and between 2-oxoglutarate isotopologue amount fractions were taken into account, although they were negligible. No other consistent correlation was found among the uncertainties.

**Figure 5:**
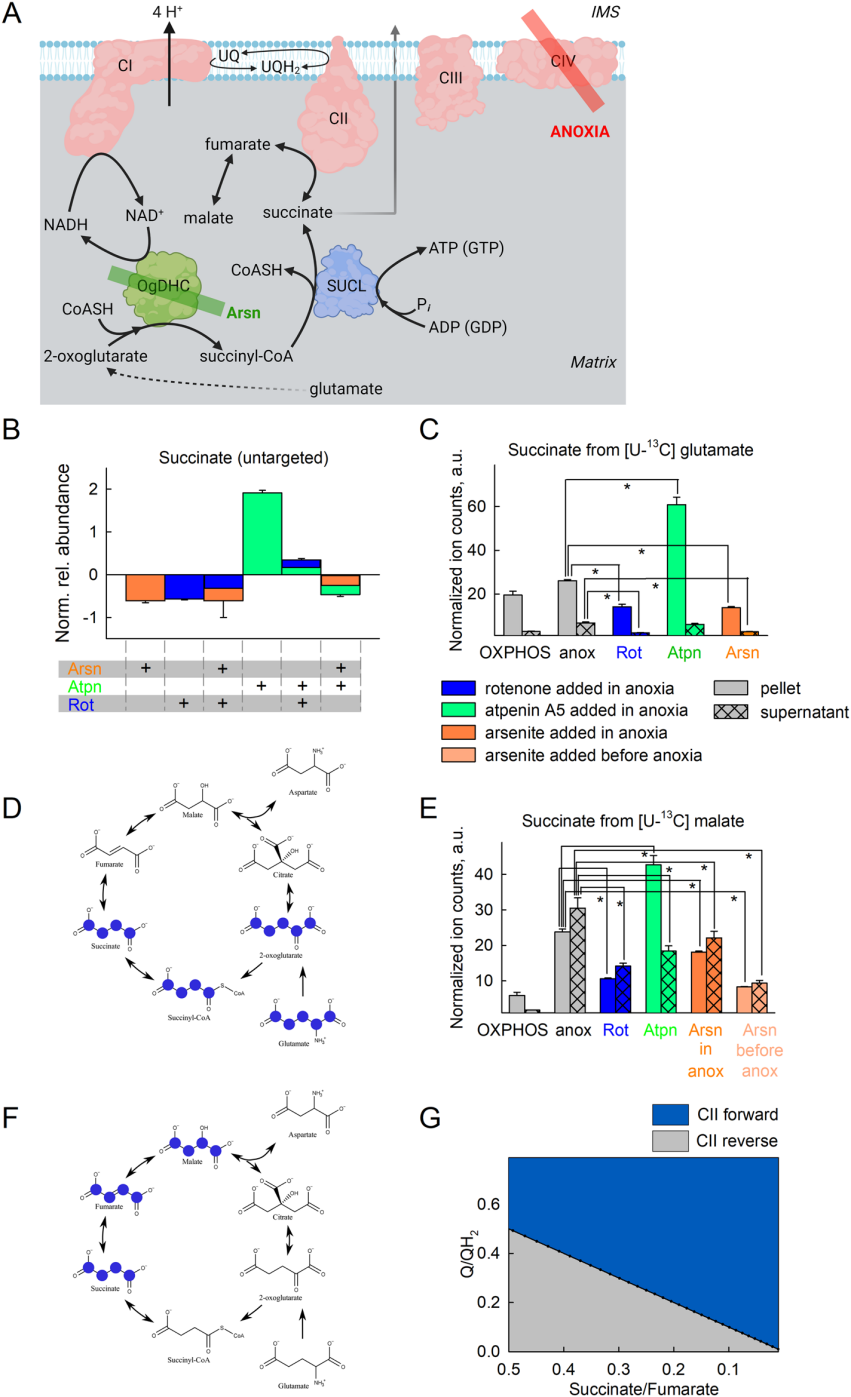
Oxidation of NADH by CI supports OgDHC, in turn maintaining the oxidative decarboxylation of 2-oxoglutarate during acute anoxia. (**A**) Scheme illustrating the action of inhibitor(s) used downstream of CI in the experiments shown in the figure (created with BioRender.com). (**B**) Untargeted metabolite analysis of succinate present in the pellets of mitochondria treated with the conditions indicated in the panel; substrates were glutamate (5 mM) and malate (5 mM). The y-axis illustrates normalized, log transformed, and scaled peak area. *Arsn (orange)* arsenite, 2 mM; *Atpn (green)* atpenin A5, 1 μM; *Rot (blue)* rotenone, 1 μM. (**C,E**) Targeted ^13^C metabolic tracer analysis of succinate present in the effluxes (supernatants) or pellets of mitochondria treated with the conditions indicated in the panel, when using [U-^13^C]glutamate (5 mM) in the presence of malate (5 mM, (**C**)) or [U-^13^C]malate (2.5 mM) in the presence of glutamate (5 mM, (**E**)). (**D,F**) Schemes illustrating the paths of ^13^C labels in glutamate or malate to succinate during anoxia, respectively. Marvin was used for drawing chemical structures, Marvin version 21.18, ChemAxon, (https://www.chemaxon.com). (**G**) 2D plot of CII directionality depicted from succinate/fumarate ratio as a function of Q/QH_2_ ratio.

Spinelli et al. reported that in hypoxia or in cells genetically modified to lack CIII or CIV, alterations in the UQH_2_/UQ ratio support the reverse operation of CII, and as a consequence of this, CI remains partially operational^41^. However, for the reasons reviewed in^57^, the reverse operation of CII is unfavored, although not precluded. In the same line of thought, the group of Brookes reported that in cardiac ischemia, succinate accumulates mostly through canonical Krebs cycle activity through which the oxidative decarboxylation of 2-oxoglutarate produced from glutamine also takes place^5^. However, they did also report that CII was also operating in reverse, albeit to a moderate extent. The conundrum of whether CII operates in forward or reverse is relevant to the present study, because the reverse operation of CII yielding UQ would essentially be the means for maintaining CI operation forming UQH_2_, especially in view of the fact that endogenous UQ pools may last only for a few seconds. We therefore examined the effect of CII inhibitors atpenin A5 (Atpn) and malonate (Mna) (Fig. 3A) added before or after the addition of CI inhibitors during anoxia, and measured the Q and NADH/NAD^+^ redox states. As shown in Fig. 3 panel B (representative trace; mean data shown in figure panel 3D and supplementary Fig. 12 panel A), addition of rotenone after any CII inhibitor in mitochondria that were previously respiring on glutamate and malate (5 mM, each) led to a greater increase in UQ implying a greater inhibition of UQ reduction than addition of CI inhibitor alone (marked by a blue dash line traversing Fig. 3D–F), in addition to conferring an increase in NADH depicted in figure panels 3G; furthermore, addition of any CII inhibitor alone during anoxia did not lead to a statistically significant increase in UQH_2_/UQ compared to vehicle. However, addition of malonate (but not atpenin A5) during anoxia led to an increase in NADH autofluorescence, see figure panel 3G. Addition of atpenin A5 after rotenone led to a greater increase in UQ, implying a greater inhibition of UQ reduction (Fig. 3E). Addition of atpenin A5 after rotenone did not lead to a statistically significant change in NADH levels during anoxia, confirming that when CI is inhibited the contribution of CII to matrix NADH levels is irrelevant, see Fig. 3H. Note that while both CI and CII operate in the direction of UQH_2_ formation, this is not masked by a potential concomitant CIII operation, oxidizing UQH_2_ to UQ; indeed, as shown in Fig. 3C (representative trace; mean data shown in figure panel 3F), the presence of the CIII inhibitor myxothiazol led to a decrease in UQ in the combined presence of rotenone and atpenin A5, implying diminished availability of UQ from CIII to CI and CII; similarly, the same can be observed in NADH levels (Fig. 3I), meaning that UQ arising from CIII would be used by CI and CII. Qualitatively similar findings were observed with the alternative CI inhibitors piericidin A and pyridaben shown in supplementary Fig. 12 A–F. Collectively, the data suggest that in acute anoxia, CII operates in the direction of UQH_2_ formation. However, these data do not argue that CII operates *exclusively* in forward mode. To address the directionality of CII during acute anoxia, we examined the effect of metabolites passing through CII by measuring Q and NADH/NAD^+^ redox states while pharmacologically inhibiting specific complexes shown in Fig. 4A. As shown in Fig. 4B, addition of succinate (S) after rotenone leads to UQ reduction under anoxia, implying that CII can still operate in forward mode. In Fig. 4C, addition of succinate before rotenone in anoxic mitochondria depicts less inhibition of UQH_2_ formation than rotenone alone (Fig. 4B), implying that succinate was impairing CI operation during anoxia. This effect of succinate was abolished by atpenin A5 (Fig. 4D). Addition of rotenone during anoxia led to an increase in NADH levels which was mitigated by the subsequent addition of succinate (Fig. 4E). Addition of rotenone after succinate during anoxia still led to an increase in NADH levels implying that CI remained operational (Fig. 4F). However, the increase in NADH levels is smaller in the presence of exogenously added succinate than in its absence (see blue dash line traversing Fig. 4E–G). Likewise, atpenin A5 abolished the effect of succinate on rotenone-induced changes on NADH levels (see Fig. 4G). Similar effects were obtained by replacing rotenone with the alternative CI inhibitors, piericidin A and pyridaben (supplementary Fig. 12G, H). These data reiterate that CII operates in forward mode in the very short initial time interval and this hinders CI’s ability to produce NAD^+^. Finally, the effect of exogenous addition of succinate in the presence of rotenone yielding a decrease in NADH levels could be interpreted by considering that CII was also operating in reverse, reducing fumarate to succinate; as such, it would generate UQ for CI and maintain a residual operation. Overwhelming the system with succinate, would mitigate this effect. This is further addressed in the subsequent section, see Fig. 5.

Next, to corroborate the finding that during acute anoxia CII operates in the direction towards succinate oxidation forming UQH_2_, we performed untargeted analysis of pertinent metabolites while ETS components where pharmacologically inhibited. Mitochondria were allowed to respire on glutamate and malate in the presence of ADP to the point of O_2_ depletion from their suspending medium. This was verified by monitoring O_2_ concentration polarographically. As shown in Fig. 4H, anoxia increases the concentration of succinate while rotenone partially abolished this. Quantification of other untargeted metabolites is shown in the supplementary Fig. 13. On the other hand, atpenin A5 not only fails to diminish succinate concentration, it even potentiates the increase. This means that during anoxia (i) CII was operating in the direction of succinate oxidation (i.e. forward mode) and that (ii) CI operation was supporting succinate formation. However, this does not exclude the possibility of CII reversal at a later time, as hypothesized above. To address this, we performed experiments on isolated mitochondria in which [U-^13^C]glutamate was added just before the onset of anoxia (when [O_2_] was ~ 10 μM) and collected them at three different time points indicated as extraction point (E.P.) 1, 2, and 3 corresponding to 76 s, + 200 s and + 400 s post-anoxia in figure panel 4I followed by quantification of selected metabolites, including ^13^C-labelled succinate. We compiled these data in a simple model in which succinate may originate from OgDHC (from 2-oxoglutarate through succinyl-CoA) or through CII reversal (from fumarate), also acknowledging a certain loss of succinate by being transported out of the matrix, depicted in figure panel 4J. The model can be formulated as:

$${\Delta }^{\text{k}}{\text{S}}=\Delta {\text{S}}_{\text{G}}\cdot {x}_{\text{G,k}}+\Delta {\text{S}}_{\text{M}}\cdot {x}_{\text{F,k}}-\Delta {\text{S}}_{\text{T}}\cdot {x}_{\text{S,k}}$$

where Δ^k^S is the total change in succinate amount of the k isotopologue; ΔS_G_, ΔS_M_, ΔS_T_ are the amounts of succinate coming from the direction of glutamate (through 2-oxoglutarate), malate (through fumarate) and transport across the inner mitochondrial membrane (IMM) respectively; *x*_G,k_, *x*_F,k_, *x*_S,k_ are the amount fractions of the k isotopologue for 2-oxoglutarate, fumarate and succinate respectively. Under k isotopologue we refer to either unlabeled (M + 0) or fully labelled (M + 5 for 2-oxoglutarate) and (M + 4 for succinate and fumarate) substances. Especially the amount fractions of 2-oxoglutarate isotopologues showed a marked change as a function of time (supplementary Fig. 14 panels A, B). To estimate the averages, we fitted an exponential and calculated the means of the functions using the coefficients of the fits. It is evident that regardless of the presence of atpenin with (blue dots) or without oligomycin (red dots) the end points of the amount fractions are nearly the same, thus it is safe to assume that the average amount fractions are nearly the same in the presence of the inhibitors as well. When atpenin A5 is present, CII is expected to be completely inhibited without affecting the rate of transport and thus we can solve for ΔS_T_ and ΔS_G_ using the above model (results shown in supplementary Fig. 14 panel E). Using this ΔS_T_ in the absence of atpenin A5 we solved for ΔS_G_ and ΔS_M_ (shown in Fig. 4 panel K) to obtain the average contribution of each pathway over the initial post-anoxic 600 s period. Seeing that the changes in matrix succinate concentration are fairly small (in arbitrary units 63.6 at 0 s, 68.8 at 200 s and 85.7 at 600 s), we assumed that this would not affect the rate of succinate transport across the IMM to a great extent, thus, we could extrapolate its value to individual time periods. This allows us to break down the gross 600 s period (Fig. 4 panel K) into constituent initial 200 s (Fig. 4 panel L) and following 400 s (Fig. 4 panel M) periods evaluated similarly. The results show that the relative contributions to succinate accumulation changes over time, from an initial OgDHC dominance (Fig. 4 panel L) to a CII reversal dominance (Fig. 4 panel M). To state this more simply, at the beginning of anoxia succinate was mostly produced using carbon atoms from glutamate, but over time succinate originates increasingly from fumarate.

### Oxidation of NADH by CI supports OgDHC, in turn maintaining the oxidative decarboxylation pathway from glutamate

To address the possibility that CI favored the formation of succinate through supporting the oxidative decarboxylation of 2-oxoglutarate at the onset of anoxia, we performed the untargeted metabolite analysis in the presence of arsenite (Arsn, HAsO_3_^2−^, see Fig. 5A) and other ETS inhibitors. When using glutamate plus malate as substrates, the only target of arsenite is OgDHC, if the variable to be measured is succinate concentration; glutamate dehydrogenase is not sensitive to arsenite^58^. Indeed, as shown in Fig. 5B, arsenite abolished the increase in succinate, irrespective of the presence of rotenone or atpenin A5. This means that succinate originated from the canonical Krebs pathway: glutamate → 2-oxoglutarate → succinyl-CoA → succinate. However, it does not mean that during anoxia, succinate originated *exclusively* from this metabolic branch, and as shown above, the contribution of succinate from CII reversal is increasing over time, while endogenous Q pools are depleted; to address this further, we traced metabolites harboring ^13^C that could originate from [U-^13^C]glutamate or [U-^13^C]malate. As shown in Fig. 5C, during anoxia the abundance of labelled succinate in mitochondria that were exogenously given [U-^13^C]glutamate is lower when rotenone was present; atpenin A5 increased the amount of labelled succinate, while arsenite decreased the abundance of succinate labelling, compared to its absence. The data obtained from ^13^C metabolic tracer analysis using [U-^13^C]glutamate agree with the untargeted metabolite analysis arguing that succinate originated from the canonical Krebs pathway, depicted in Fig. 5D. It is noteworthy that in the presence of atpenin A5, while succinate is building up significantly, it is unable to exit mitochondria as easily as under other conditions (ratio of pellet to supernatant is significantly altered). The reason(s) behind this could be due to enhanced malate uptake or excretion of another dicarboxylate, effectively out-competing succinate; alternatively, this could be a result of issues co-transporting inorganic phosphate (P_*i*_): the dicarboxylate transporter can exchange a dicarboxylate for P_*i*_^59^, so when P_*i*_ builds up in the matrix (due to extensive ATP hydrolysis by a reverse-operating F_1_F_O_-ATPase^35^) the exchange for succinate could be inhibited, hence malate enters against citrate and the dicarboxylate carrier is effectively fully inhibited. By inhibiting ATP hydrolysis with oligomycin, succinate export was affected but not to a significant extent (supplementary figure panel 14F), although this could be confounded by other factor(s), such as depolarization of mitochondria. This line of thought was not pursued further. The abundance of other targeted metabolites is shown in supplementary Fig. 15. There, the ~ 5-fold increase of oxoglutarate from [U-^13^C]glutamate by arsenite indicates genuine inhibition of the oxoglutarate Complex. Results on labeled fumarate also confirm that while fumarate hydratase is working at equilibrium loading the pool from unlabeled malate, contribution from glutamate is significantly reduced after addition of rotenone, atpenin A5 and arsenite. The reduction after rotenone is similar to that of succinate, showing the reduction is upstream, as opposed to the presence of atpenin A5. However, when using [U-^13^C]malate, there was succinate labelling during anoxia (Fig. 5E) but with no labelling of oxoglutarate (supplementary Fig. 16A). This could only mean that CII was also operating in reverse to some extent, depicted in Fig. 5F, and consistent with the notion that most of the citrate being produced is being exported against the malate gradient, essentially reinforcing the conclusions obtained from the data and model shown in figure panels 4I-4M. The abundance of other metabolites that acquired labelling from [U-^13^C]malate is shown in supplementary Fig. 15. Regarding Fig. 5D, F even though the reaction catalyzing the interconversion of malate to oxaloacetate by malate dehydrogenase is definitely occurring as evidenced by the aspartate labeling, oxaloacetate is omitted as we could not detect it. In Fig. 5D, F we only mark metabolites outlining the metabolic routes taken, and not all metabolites detected with the ^13^C labels, for clarity.

So how can it be that CII operates amphidirectionally? The reaction catalyzed by CII is succinate + UQ ↔ fumarate + UQH_2_; however, there are other reactions affecting each reactant as well: succinate is also influenced by succinyl-CoA ligase, fumarate by fumarase, UQ and UQH_2_ by CI and other enzymes converging at the mitochondrial coenzyme Q junction^30,31^. By plotting succinate/fumarate ratio (on the basis of metabolite content in the pellets during anoxia, obtained from the targeted ^13^C metabolic tracer analysis) vs UQ/UQH_2_ ratio, it is apparent that CII directionality is dictated by the pair of values across a straight line, see Fig. 5G. However, fumarase, CI, and enzymes converging at the mitochondrial Q junction (as well as other enzymes having succinate or fumarate as substrates or products, see https://metabolicatlas.org/explore/Mouse-GEM/gem-browser/metabolite/MAM02943m and https://metabolicatlas.org/explore/Mouse-GEM/gem-browser/metabolite/MAM01862m, respectively) will lead to a shifting pattern of succinate/fumarate and UQ/UQH_2_ pair values across this line, which means that the direction favored by CII will be changing as dictated by the very same value pairs. In addition, since UQ may be protein-bound or unbound to the extent of 10–32%^45^ the amount of protein-unbound UQ and the parameters influencing this binding (such as the lipophilicity property of the UQ type^60–62^) will also dictate CII directionality.

Mindful that succinate may arise from either (i) glutamate → 2-oxoglutarate → succinyl-CoA → succinate and/or (ii) reduction of fumarate implying CII reversal, the question arises which pathway contributes more significantly to altering succinate concentration in the matrix during acute anoxia. To this end, we titrated the amount of exogenously added succinate in reversing ANT directionality during anoxia; the rationale of this approach is that if CII reversal is considerable, the amount of succinate generated should inhibit the ATP-forming mtSLP which dictates ANT directionality^12^. As shown in supplementary Fig. 16B the concentration of exogenously added succinate for causing CAT-induced delayed loss of Δ*Ψ*_mt_ after anoxia is in the order of 0.5–2 mM (left three panels). The *K*_*m*_ of the dicarboxylate transporter translocating succinate across the mitochondrial inner membrane is > 1.1 mM^63^. Accumulation of succinate in the matrix is also dependent on Δ*Ψ*_mt_; the more depolarized mitochondria are, the less succinate is taken up^64^. It is therefore reasonable to assume that addition of 0.5 mM succinate to already depolarized mitochondria due to anoxia leads to a small increase in matrix succinate concentration, well below 0.5 mM. As this is a small amount, we conclude that CII could not operate in reverse to an appreciable degree during the very first moments of acute anoxia, as the amount of succinate formed is insufficient to hinder mtSLP. Furthermore, exogenously added 0.5 mM succinate still yielded UQ reduction to UQH_2_, implying that CII was mostly operating in forward mode (figure supplementary Fig. 16B, rightmost panel). However, as the endogenous Q pools become rapidly reduced over time commencing from the post-anoxic event, the contribution of succinate from CII reversal becomes more and more significant, for as long as the Q pools are not re-oxidized.

**Figure 6:**
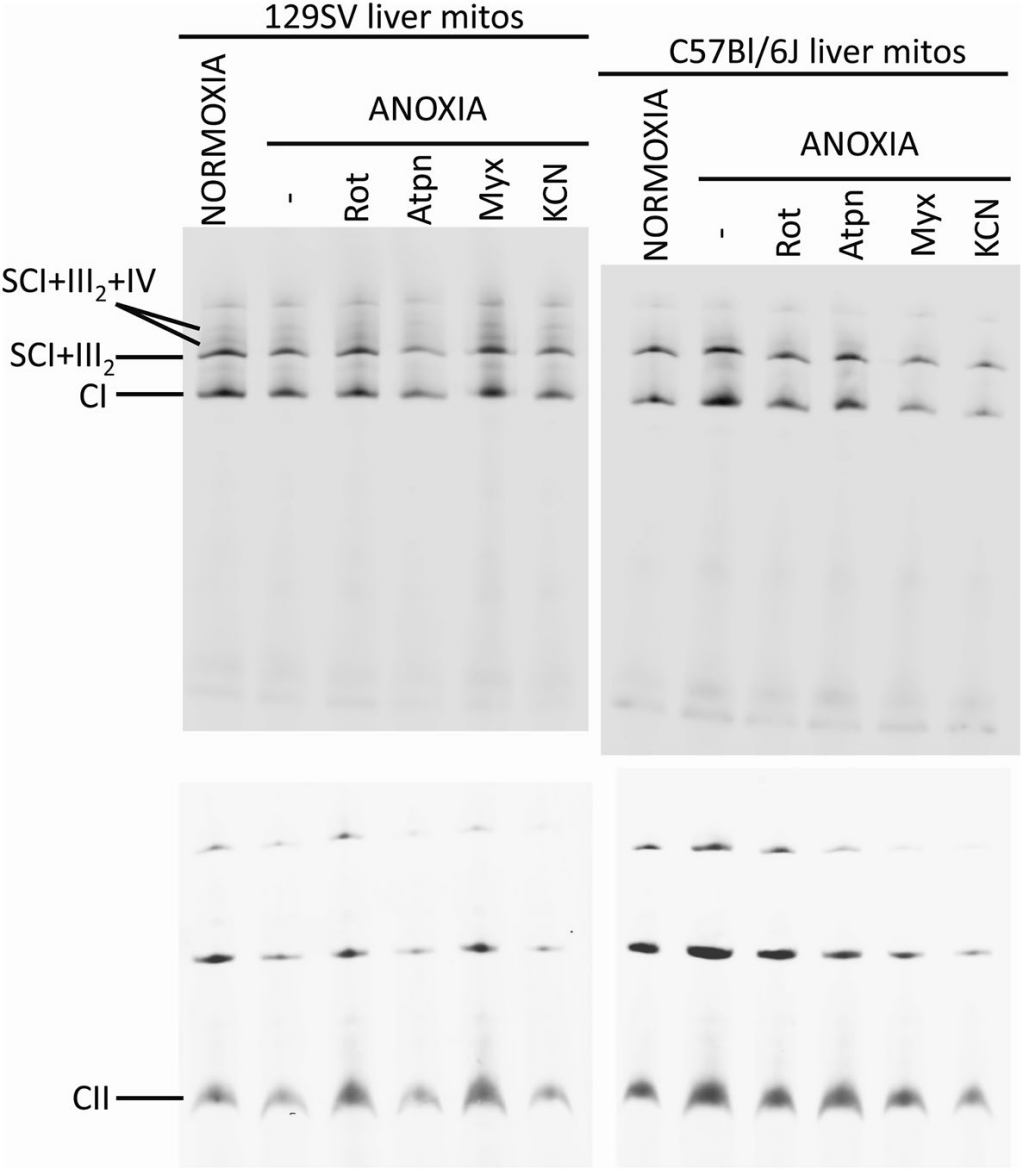
Acute anoxia and targeted inhibition of the ETS exert no impact on the stability of complex I or on the formation of supercomplexes. Western blot and immunodetection of Blue-Native electrophoresis gels. The formation of respiratory supercomplexes containing CI was determined in liver mitochondria from 129/Sv and from C57Bl/6J mice in normoxia and in either anoxia alone or combined with treatment with different inhibitors (*Rot* rotenone; *Atpn* atpenin A5; *Myx* myxothiazol; *KCN* potassium cyanide). The main bands detected correspond to free complex I (CI) and to the supercomplex formed by the association of complexes I and dimeric III (SC I+III_2_). The higher molecular bands of lower intensity correspond to the association of complexes I, dimeric III and IV, also known as the ‘respirasomes’ (SC I+ I+III_2_+IV). Complex II (CII), migrating as a single entity of approximately 140 kDa, was also immunodetected in the same samples, to serve as a loading control.

Finally, in view of the proposal that hypoxia modulates respiratory chain supercomplex formation resulting in the stabilization of these supramolecular structures^65,66^, we tested whether conditions of acute anoxia had an impact on the stability of Complex I and on the formation of supercomplexes. The isolated mitochondria that were either kept in normoxia or subjected to acute anoxia with or without inhibitor treatments were solubilized using digitonin, a detergent that maintains the interaction between the complexes, and run through Blue-Native Gel Electrophoresis (BNGE). As shown in Fig. 6, the immunodetection of Complex I revealed no major changes either in supercomplex distribution or in the levels of any of the Complex I-containing species (free Complex I and supercomplexes I + III_2_ and I + III_2_ + IV–respirasomes) induced by acute anoxia using NDUFS1- and SDHB antibody for complexes I and II, respectively. This was tested not only in liver mitochondria from C57Bl/6J mice, which due to the absence of a functional Cox7a2l showing a different supercomplex pattern^67,68^ but also in mitochondria from the 129/Sv mouse strain in which all the supercomplex species can assemble.

## Discussion

It is a textbook definition, that -except in animal species adapted to prolonged survival under anoxia^69^—in the absence of oxygen there is no electron transport in the mitochondrial respiratory chain, the Krebs cycle is inhibited, and there is accumulation of reducing equivalents in the mitochondrial matrix. However, the catabolism of glutamine by the oxidative decarboxylation route entering the Krebs cycle during hypoxia has been unequivocally demonstrated^5,35,70,71^; the significance of this echoes the need for addressing the reductive stress when combating diseases spanning from ischemia–reperfusion to cancer^4^. What has not been fully addressed is the origin of NAD^+^ for supporting OgDHC in the pathway glutamine → glutamate → 2-oxoglutarate → succinyl-CoA → succinate, for the 2-oxoglutarate to succinyl-CoA conversion during anoxia. To this end, various potential sources have been theorized to contribute^72^, including CI. Indeed, in cells experiencing hypoxia (1% O_2_) or genetically engineered to lack key components of either Complex IV (CIV) or Complex III (CIII) rendering them incapable of using O_2_ as a terminal electron acceptor, CI was still able to deposit electrons into the ETS^41^. The experiments of Spinelli et al. on generating such cell lines was instrumental in addressing the concept that CIV exhibits a sufficiently high affinity for O_2_ that could maintain full activity even in 1% O_2_^73^. Spinelli et al.^41^ proved that under such conditions succinate dehydrogenase (CII) was operating in reverse reducing fumarate, supported by the high UQH_2_/UQ ratio. In their work^41^, residual activity of CI in hypoxia or in cells lacking CIII or CIV was implied, something that has been previously proposed as plausible by mathematical modelling, even in anoxia^72^. On the same line of thought, the group of Finch showed that NAD^+^ originating from the reverse operation of the mitochondrial malate dehydrogenase isoform 2 (MDH2) support glutamate dehydrogenase (GDH) activity, fulfilling anaplerosis in respiration-deficient cells^74^. Likewise, the group of Tennant showed that in hypoxic conditions mitochondrial pyrroline 5-carboxylate reductase 1 (PYCR1) activity is increased yielding NAD^+^, permitting continued TCA cycle activity in vivo and in 3D cultures^75^.

The most important finding of the present study is that during anoxia CI remains operational, albeit to a diminished, but sufficient extent for supporting the reaction catalyzed by OgDHC with NAD^+^. The significance of this finding has the following four ramifications:

(i) It can explain earlier findings reporting that CI inhibitors yield less succinate when added in hypoxic tissues ^5,76,77^. The accumulation of succinate in ischemia is unquestionable ^78–81^, however, its origin is debated; on one hand, CII reversal reducing fumarate to succinate yielding UQ for CI has been convincingly demonstrated ^41,78^; on the other, succinate formation by the oxidative deamination of glutamine/glutamate and following canonical Krebs cycle activity has been shown to be quantitatively more important ^5^. Here we show that during the anoxic event CII was functional and its directionality changed from forward to reverse, most likely due to the rapidly declining sources of endogenous quinones. In either case -CII reversal yielding succinate from fumarate or CII forward diverting UQ away from CI- CI remains operational in anoxia, providing OgDHC with NAD^+^. Relevant to this, the finding that CII reversal is critical for maintenance of CI operation could explain our earlier findings on comparing the effect of 2-oxoglutarate in the presence and absence of malate, on mtSLP during either CI inhibition by rotenone vs in anoxia ^82^. As shown in supplementary Fig. 18 (reproduced by permission; it is an excerpt of Fig. 5 of reference ^82^) the presence of malate (when 2-oxoglutarate is already present) is able to rescue mtSLP in anoxic mitochondria, i.e. 2-oxoglutarate alone is not sufficient. This is because malate is able to follow the route of malate → fumarate → succinate. The latter part of this pathway entails CII in reverse, providing UQ (from UQH_2_) to CI, which generates NAD^+^, donates it to OgDHC and mtSLP is maintained. Of note, malate becoming oxaloacetate could not be the reason for this rescue, because: (i) when respiratory chain is deficient, the mitochondrial malate dehydrogenase 2 operates in reverse, oxidizing NADH ^74^ and (ii) oxaloacetate would inhibit succinate dehydrogenase that in turn would disfavor CII reversal. The ability of the tissue to withstand anoxia will eventually depend on the availability of Q pools for the acute phase (for a few seconds) and the availability of fumarate (which could originate from malate) for the latent phase. Fumarate supporting CI and in extension of this both Δ*Ψ*_mt_^83^ and provision of NAD^+^ to OgDHC (this study) could arise from aspartate through the concerted action of purine nucleotide cycle and aspartate aminotransferase ^78^. However, there are two concerns with this concept: first, the purine nucleotide cycle is an energy-dependent process ^84^; second, the energy provided by CI could not be in the form of Δ*Ψ*_mt_ to a sufficient extent: acknowledging that CI activity in anoxia is ~ 10% of its theoretical maximum and mindful that only 4 protons are pumped out of the 4 + 4 + 2 for CI, CIII and CIV, respectively by the ETS and that Δ*Ψ*_mt_ fluctuates between −108 and −158 mV in normoxia ^85^ and is clamped to ~ −100 mV in anoxia^35^, the contribution of CI to generating membrane potential in anoxia should be a meager 2.5—3.7 mV, which is reflected in our rhodamine 123 recordings, given the evanescent changes in fluorescence. Thus, it is likely that the availability of UQ for CI for the acute phase shown hereby may generate sufficient UQH_2_ that can support CII reversal in the latent phase.
(ii) Oxidation of UQH_2_ in mitochondria is necessary for tumor growth ^86^, where oxygen availability is frequently limited ^87^: it is extremely likely that NADH oxidation by CI requiring UQ in hypoxia is the missing link for providing NAD^+^ to OgDHC supporting the oxidative decarboxylation branch of glutaminolysis; glutaminolysis is a hallmark of many cancers ^88^. Indeed, the group of Chandel ^86^ demonstrated that the loss of CI thwarted tumor growth and this was rescued by mitochondrially-targeted expression of the NADH oxidase LbNOX ^89^.
(iii) Relevant to the results published in ^86^, the role of CI in the proliferation of cancer cells has been reviewed in ^90^, and the consensus was that a complete- as opposed to an incomplete- loss of CI activity halts tumor growth, although avoidance of reactive oxygen species (ROS) generation was proposed to mediate this effect. In addition, CI inhibitors exhibiting potency against solid cancers in murine models and human cell lines are being evaluated in > 400 clinical trials ^91^.
(iv) CI has been described to exist in two forms, an active (*A*) and a de-active (*D*) form, the latter signifying a dormant state of the complex which is not inactivated or covalently modified in any way ^92^; the *A* to *D* transition occurs during ischemia, i.e. when substrate and oxygen availability are limited ^93^. The *D* form is thought to exert a protective role by delaying the rate of regaining normal respiration rates during reoxygenation, thus potentially mitigating ROS production ^94^; the other side of the coin is that when in *D* form, CI risks becoming permanently inactive ^95^. Although it is not possible to decipher if residual CI activity maintained by UQ availability during acute anoxia will be beneficial or not regarding ROS formation *vs* risk of permanent inactivation, it certainly influences the *A* to *D* transition phenomenon.

A common denominator of the above is that subsequent catabolism of succinyl-CoA, the product of OgDHC which is supported with NAD^+^ provided by residual CI activity during anoxia will contribute to mtSLP. The generation of high-energy nucleotides by mtSLP has been unjustly side-lined in the literature; it is true that the rate of ATP (or GTP, depending on subunit composition of the succinyl-CoA ligase and the presence of a nucleoside diphosphokinase^96,97^) synthesis dwarfs compared to that by the mitochondrial F_1_F_O_-ATPase. However, during anoxia the F_1_F_O_-ATPase is hydrolyzing ATP instead of producing it^98^; in this case, the amount of ATP produced by mtSLP in combination to the much smaller volume of mitochondrial network compared to that of the cytosol amplifies the effects of ATP production in terms of concentration. This could rescue a tissue experiencing respiratory inhibition by preventing mitochondria from becoming ATP drains^36^.

In conclusion, we hereby showed that in isolated murine mitochondria experiencing anoxia, CI exhibits sufficient activity yielding NAD^+^ for OgDHC that in turn forms succinyl-CoA supporting mtSLP; the availability of UQ is a critical factor for this residual CI activity. Forward operation of CII is necessary for maintaining succinate levels low enough to allow the reversible succinyl-CoA ligase reaction to proceed towards ATP (or GTP) synthesis, but on the other hand, CII reversal also occurs reducing fumarate to succinate and oxidizing UQH_2_ to UQ, the latter favoring CI activity. All other enzymatic reactions in which succinate, fumarate, UQ and UQH_2_ participate, contribute to CII directionality and ultimately influence CI residual activity.

### Limitations of present study

A main concept of the present work is the demonstration that UQ availability dictates CI activity; therefore, manipulation of Q pools by disrupting CoQ biosynthetic pathways could have been argued to lend strong support to the above claim. However, disrupting CoQ biosynthesis leads to impaired mitochondria^99^; it would be exceedingly difficult to achieve anoxic state in impaired mitochondria due to negligible OXPHOS state of respiration. Thus, we have alternatively relied on limiting vitamin K supplementation to mice; “vitamin K” is actually a group of naphthoquinones that includes menaquinone and phylloquinone^100^. These two quinones carry electrons in bacteria and plants, respectively, whereas eukaryotes use ubiquinone (CoQ). However, the eukaryotic enzyme ferroptosis suppressor protein 1 (FSP1), a NAD(P)H-ubiquinone reductase reduces both vitamin K quinones and CoQ^101^ and by doing this, vitamin K may “spare” CoQ pools. Since vitamin K is a regular dietary constituent, we reasoned that by decreasing vitamin K availability in a relatively acute manner (mice were subject to a vitamin K-deficient diet for 1–3 weeks) CoQ pools could be sufficiently diminished but not to the extent of impairing OXPHOS respiration.

Another limitation is that electrochemical, real-time detection of mitochondrial quinone pools cannot be performed in intact, living cells using Q_2_ (or any other) mediator, due to ‘enclosure’ of mitochondria by the cytosol and plasma membrane. Thus, it is not possible to address in situ CI activity by recording changes in Q levels using the three-electrode system in intact, living cells.

An additional limitation is that mouse liver mitochondrial metabolism differs from other tissues^102^; to this end, it was shown that CI is dispensable in the adult murine liver. Although this specific aspect does not necessarily diminish the importance or credibility of the present work, it must not be ignored that there could be some other difference downstream from CI just as major as the dispensability of this ETS complex for mouse survival that could confound our interpretations. Relevant to this, our main findings were reproduced in mitochondria isolated from brains and hearts of mice. Finally, the presence of quinone binding sites on CI that are rotenone-insensitive may also confound our interpretations on the effects of this inhibitor on Q changes^103–105^ reviewed by Lenaz^62^.

### Supplementary Information

Supplementary Figures.

Supplementary Table 1.

## Data Availability

All data have been deposited at Mendeley (doi: 10.17632/5tj4kymggt.1) and are publicly available as of the date of publication. This paper does not report original code. Any additional information required to reanalyze the data reported in this paper is available from the lead contact (Christos Chinopoulos; Email: chinopoulos.christos@semmelweis.hu) upon request.
