# Supplementary material for: Residual Complex I activity and amphidirectional Complex II operation support glutamate catabolism through mtSLP in anoxia
Source: Sci Rep. 2024 Jan 19;14:1729. doi: 10.1038/s41598-024-51365-4 (PMC10798963; doi:10.1038/s41598-024-51365-4)
Supplement: Supplementary file 1 — Supplementary Figures. [file 41598_2024_51365_MOESM1_ESM.pdf]

SUPPLEMENTARY MATERIAL

Legend to supplementary figure 1: A, B: oxygen concentration (top panels, in μM) recorded simultaneously with UQ redox state (middle panels) and rhodamine 123 fluorescence (Rhod123, bottom panels) in isolated mouse liver mitochondria; panels are aligned in the x-axis. C: Quantification of the vehicle or CI-inhibitor Pyridaben (Prdb) or Piericidin A (Pierc) induced changes in Q signal in anoxic mitochondria, as illustrated by the bidirectional arrows in main figure 1C (middle panel). D: NADH autofluorescence in isolated mouse liver mitochondria. Mitochondria (mito), ADP (2 mM), rotenone (Rot, 1 μM), carboxyatractyloside (CAT, 1 μM), SF (SF6847, 0.25 μM) were added where indicated. Substrates were glutamate and malate (5 mM each) present in the buffer prior to addition of mitochondria. E: Effect of anoxia on NADH autofluorescence in isolated mouse liver mitochondria. F: Quantification of the vehicle or CI-inhibitor induced changes in NADH signal in anoxic mitochondria.

Legend to supplementary figure 2: A-G: Schematics (A) and pictures (B-G) of the air-tight glass chamber with the glass lid and septum, pierced by a Hamilton syringe. This chamber is designed to fit the cuvette holder of a Hitachi F-7000 spectrofluorimeter. In the bottom of the chamber, a magnetic stirrer is in place. H, I: NADH exc/em spectra (black *vs* red traces) acquired in a polymethacrylate cuvette (H) and in the glass chamber (I), respectively. J: Representative rhodamine 123 fluorescence traces in isolated mouse liver mitochondria suspended in the glass chamber undergoing anoxia, and the effect of rotenone (Rot, trace b) vs vehicle (veh, trace a) in altering the CAT-induced change in mitochondrial membrane potential. K, L: Representative NADH autofluorescence traces in isolated mouse liver mitochondria suspended in the glass chamber undergoing anoxia, and the effect of vehicle (veh) (K) vs rotenone (Rot) (L) in altering the CAT-induced change in mitochondrial membrane potential. M, N: Representative NADH autofluorescence traces in isolated mouse liver mitochondria suspended in the glass chamber undergoing anoxia in the presence of either Myxothiazol (Myx) or succinate (S) added where indicated. O, P: Quantification of the Rot-induced changes in NADH autofluorescence as a function of the inhibitors/metabolites added as indicated in the bottom of the panel. Data pooled from 4 independent experiments. * $p<0.05$. Note that the presence of succinate (5 mM) abolishes the Rot-induced increase in NADH signal in anoxia.

Legend to supplementary figure 3: A, B, C: oxygen concentration (top panels, in μM) recorded simultaneously with rhodamine 123 fluorescence (Rhod123, bottom panels) in isolated mouse liver mitochondria, simultaneously to NADH autofluorescence (shown in supplementary figure 1D, supplementary figure 1E and main figure 1E); panels are aligned in the x-axis. Note that in A, B, C the presence of rhodamine 123 decreased slightly OXPHOS respiration rate (compare black with red traces), thus anoxia commenced at slightly different times. This is similar to what has been reported for safranine O [1], [2] but is quantitatively much less pronounced. D-G: Oxygen concentration (top panels, in μM) recorded simultaneously with either Q redox state (D, E, middle panels) or NADH autofluorescence (F, G middle panels) and/or rhodamine 123 fluorescence (Rhod123, bottom panels) in isolated mouse liver mitochondria. Substrates were glutamate and malate (5 mM each) present in the buffer prior to addition of mitochondria. Rhodamine 123 fluorescence indicative of $\Delta\Psi_{mt}$ (arbitrary units, a.u.) was recorded separately from NADH autofluorescence due to spectral overlap. Mitochondria (mito), ADP (2 mM), rotenone (Rot, 1 μM, panel A, C), vehicle (veh, panel B), pyridaben (Prdb, 1 μM, panel D, F), or piericidin A (Pierc 1 μM, panel E, G), carboxyatractyloside (CAT, 1 μM, all panels), SF (SF6847, 0.25 μM, all panels), were added where indicated. H: Experiments are identical as shown for panel G, but atpenin A5 (Atpn, 1 μM) was added *in lieu* of a CI inhibitor.

Legend to supplementary figure 4: A: Oxygen concentration (dotted lines, in μM) recorded simultaneously with rhodamine 123 fluorescence (Rhod123, a.u.) in isolated mouse brain mitochondria undergoing anoxia and the effect of rotenone (Rot, trace b) vs vehicle (veh, trace a) in altering the CAT-induced change in mitochondrial membrane potential. B: Exact conditions as shown

for panel A, but brain mitochondria were suspended in the air-tight glass chamber. C, D: Identical experiments as shown in panels A, B, but recording NADH autofluorescence in isolated mouse brain mitochondria instead of Rhodamine 123 fluorescence. E: Quantification of the vehicle or rotenone induced changes in NADH signal in anoxic mitochondria (n=4).

Legend to supplementary figure 5: A: Oxygen concentration (dotted lines, in μM) recorded simultaneously with rhodamine 123 fluorescence (Rhod123, a.u.) in isolated mouse heart mitochondria undergoing anoxia and the effect of rotenone (Rot, trace b) vs vehicle (veh, trace a) in altering the CAT-induced change in mitochondrial membrane potential. B: Exact conditions as shown for panel A, but heart mitochondria were suspended in the air-tight glass chamber. C, D: Identical experiments as shown in panels A, B, but recording NADH autofluorescence in isolated mouse heart mitochondria instead of Rhodamine 123 fluorescence. E: Quantification of the vehicle or rotenone induced changes in NADH signal in anoxic mitochondria (n=4).

Legend to supplementary figure 6: Directionality of CI operation: forward *vs* reverse operation (the latter is represented by the shaded area) as a function of QH_2/Q, $NAD^+/NADH$, matrix pH (pH_{in}), $\Delta\Psi_{mt}$ and ΔpH across the inner mitochondrial membrane and highlighted area indicating plausible conditions of anoxia in isolated mouse liver mitochondria. Panels to the left share the same y-axis with those corresponding to the right. All panels share the same x-axis.

Legend to supplementary figure 7: A, B: Light scatter (520/520 exc/em) of mouse liver mitochondria indicative of swelling; in A, experiments were identical to those performed in main figure 1C, E; the ETS inhibitor is indicated in the panel. Rotenone (Rot, 1 μM), vehicle (veh), pyridaben (Prdb, 1 μM), atpenin A5 (Atpn, 1 μM), stigmatellin (Stigm, 0.5 μM), carboxyatractyloside (CAT, 1 μM), alamethicin (40 μg). In B, $CaCl_2$ pulses (0.3 mM) are added where indicated to demonstrate the extent of swelling that these mitochondria can reveal. Substrates were glutamate and malate (5 mM each) present in the buffer prior to addition of mitochondria. Experiments on light scatter were performed using a standard cuvette-based spectrofluorimeter (Hitachi F-7000) plugged with a custom-made plug for polymethacrylate cuvettes by 3D-printing, depicted in panel C. The plug 3D design and instructions for use are published in https://www.thingiverse.com/thing:3156148.

Legend to supplementary figure 8: A-G: Representative traces of the CI inhibitor-induced change in Q redox state signal (% scale); data pooled from such traces are shown in supplementary figure 9-A-E. For panels A, D, E, F and G the CI inhibitor is rotenone (Rot, 1 μM); for B, pyridaben (Prdb, 1 μM); for C, piericidin A (Pierc, 1 μM). Substrates are indicated in the panels (G: glutamate; Og: oxoglutarate; M: malate; all at 5 mM). H: PT time in mice fed regular *vs* vitamin K_3-deficient diet for 3 weeks; I: total quinones extracted from the livers of mice fed regular diet *vs* vitamin K_3-deficient diet; J: quantification of the rotenone (Rot) induced changes in Q signal from mitochondria obtained from mice fed regular- *vs* vitamin K_3-deficient diet; veh: vehicle (for rotenone, i.e. ethanol). * p<0.05. K, L: quantification of the vehicle- (veh, grey) *vs* rotenone- (Rot, blue) induced changes in Q signal (K) or NADH signal (L) from anoxic liver mitochondria isolated from 6N (smooth) vs 6J (cross-hatched) mice. Data pooled from 4-5 independent experiments. N.S.: not significant.

Legend to supplementary figure 9: A-E: The CI inhibitor-induced change in UQ signal (% scale) plotted as a function of time elapsed from the onset of anoxia until the addition of CI inhibitors (time in anoxia, sec). For panels A, B, C, the CI inhibitor is rotenone (Rot, 1 μM); for D, pyridaben (Prdb, 1 μM); for E, piericidin A (Pierc, 1 μM). Substrates are indicated in the panels (G: glutamate; Og: oxoglutarate; M: malate; all at 5 mM). F: Representative trace of permeabilized (using alamethicin, 20 μg, added where indicated) mitochondria to oxidize exogenously added NADH (100 μM) during anoxia, and the effect of rotenone (Rot, 1 μM), in the absence of exogenously added quinones. G: quantification of NADH-oxidizing activity calculated from traces as shown in panel F. The rotenone-

sensitive portion of NADH oxidizing activity is shown in blue and the rotenone-insensitive in violet, in the stacked bars. Violet asterisks mark a statistically significant difference between total NADH-oxidizing activities; blue asterisks indicate a significant difference between rotenone-sensitive activities. Data for bar graph G were obtained in the absence of exogenously added quinones. Data pooled from 3-9 independent experiments. * $p<0.05$.

Legend to supplementary figure 10: A:, C: Repre–sentative traces in NADH autofluorescence in mouse liver mitochondria treated by KCN (A) or true anoxia (C) and the effect of rotenone (Rot). In trace A, Q1 (40 μM), NADH (100 μM) and 5 mM succinate were present. Mitochondria were permeabilized by alamethicin(20 μg) . In trace C, fumarate (Fum) was added where indicated. B, D: quantification of the rotenone-sensitive changes in NADH autofluorescence from data pooled from 4 independent experiments, performed as in panel A, C. Fumarate/Succinate (Fum/Succ) ratio was altered as indicated in the panels.

Legend to supplementary figure 11: Quantification of inhibitor-induced changes in Q (A) or $NADH/NAD^+$ (B) redox state. Substrates were glutamate and malate (5 mM each) present in the buffer prior to addition of mitochondria. Vehicles or inhibitors present as indicated by the plus (+) signs at the bottom of the panels. Eth: ethanol (grey); wat: water (dark grey); Myx: myxothiazol, 0.1 μM (orange); Prdb: pyridaben, 1 μM (cyan); Pierc: piericidin A, 1 μM (light blue). The additions of the compounds mentioned in the bottom of each panel were made from top to bottom. Data pooled from 3-34 independent experiments. * $p<0.05$. Whenever two or three crosses are indicated within a single box in the bottom of a panel mentioning additions of chemicals, they signify double or triple addition of the vehicle (to match the number of additions to an equal number of additions of inhibitor types).

Legend to supplementary figure 12: Quantification of inhibitor-induced changes in Q (A, B, C, G) or $NADH/NAD^+$ (D, E, F, H) redox state. Substrates were glutamate and malate (5 mM each) present in the buffer prior to addition of mitochondria. Rot, (blue) 1 μM: Rotenone; Mna 5 mM (dark green): malonate; Atpn, 1 μM (green): atpenin A5; Myx 0.1 μM (orange): myxothiazol; Prdb, 1 μM (cyan): pyridaben; Pierc, 1 μM (light blue): piericidin A. The additions of the compounds mentioned in the bottom of each panel were made from top to bottom. In panels G and H, succinate (S, 5 mM) was added where indicated. Data pooled from 3-34 independent experiments. * $p<0.05$.

Legend to supplementary figure 13: Analysis of untargeted metabolites present in the pellets of mitochondria treated with the conditions indicated in the panel. The y-axis illustrates normalized, log transformed, and scaled peak area. ADP, 2 mM, S: succinate, 5 mM; P: pyruvate, 5 mM; G: glutamate, 5 mM; M: malate, 5 mM; Rot (blue): rotenone, 1 μM; Atpn, 1 μM (green): atpenin A5; Stigm, 0.5 μM (light red): stigmatellin; CN, 1 mM (yellow): cyanide; Arsn, 2 mM (orange): arsenite.

Legend to supplementary figure 14: Changes in isotopologue composition over time. Symbols are black without inhibitor, red with atpenin, blue with atpenin and oligomycin. Blue and red are slightly offset at 600s from the black symbols to reduce crowding. The line represents the fitted exponential curve. A, 2-oxoglutarate M+5; B, 2-oxoglutarate M+0; C, succinate M+0; D, succinate M+4. E, F: Average rates of succinate formation and transport expressed as velocity (ν). Magenta signifies succinate from glutamate and green succinate transported across IMM. Panel E in the presence of atpenin; Panel F in the presence of atpenin and oligomycin. For panels E and F: combined standard uncertainty was calculated using Gauss' law of error propagation with linear approximation using the NIST uncertainty machine v1.5. Subsequently, covariance between succinate isotopologue amount fractions and between 2-oxoglutarate isotopologue amount fractions were taken into account, although the correction was negligible. No other consistent correlation was found among the uncertainties.

Legend to supplementary figure 15: ^{13}C-tracer analysis of metabolites present in the effluxes (supernatants) or pellets of mitochondria treated with the conditions indicated in the x-axis, when using [U-^{13}C]glutamate (5 mM) or [U-^{13}C]malate (2.5 mM). Rot, 1 μM (blue): rotenone; Atpn, 1 μM (green): atpenin A5; Arsn, 2 mM (orange): arsenite.

Legend to supplementary figure 16: A: Labelling in oxoglutarate from [U-^{13}C]malate (2.5 mM) in the presence of glutamate (5 mM) present in the effluxes (supernatants) or pellets of mitochondria treated with the conditions indicated in the panel. B: Succinate (S, 0.5, 1, 2 mM) diminishes the CAT-induced changes in rhodamine 123 fluorescence (solid lines, Rhod123, arbitrary units, three leftmost subpanels) in anoxic mouse liver mitochondria (oxygen concentration in μM, shown in dotted lines) in addition to reducing UQ to UQH_2 (rightmost subpanel). Data pooled from 3-6 independent experiments. * $p<0.05$.

Legend to supplementary figure 17: Cyclic voltammograms in the absence (black traces) or presence (red traces) of chemicals used in this study. Rhod123: rhodamine 123 (5 μM), Rot: rotenone (1 μM), Prdb: pyridaben (1 μM), Pierc (1 μM): piericidin, Atpn: atpenin A5 (1 μM), Mna: malonate (5 mM), Myx: myxothiazol (0.1 μM), CAT: carboxyatractyloside (1 μM), SF: SF6847 (0.25 μM).

Legend to supplementary figure 18: Mitochondrial substrate-level phosphorylation in CI-inhibited using rotenone (Rot) (A, B) vs anoxic (C, D) mitochondria as a function of either Og+malate (A, C) or only Og (B, D). Panels show reconstructed time courses of safranine O signal calibrated to $\Delta\Psi m$ in isolated mouse liver mitochondria alone (A, B) or simultaneous to oxygen concentration (dotted lines, in μM), (C, D). ADP (2 mM) rotenone (Rot, 1 μM), carboxyatractyloside (CAT, 1 μM), SF (SF6847, 0.25 μM were added where indicated. Respiration substrates used are shown in the panels. Their concentrations were: malate (mal): 5 mM , 2-oxoglutarate (Og): 5 mM. Reproduced by permission from [3].

REFERENCES CITED

1. Valle, V. G., Pereira-da-Silva, L. & Vercesi, A. E. Undesirable feature of safranine as a probe for mitochondrial membrane potential. *Biochem Biophys Res Commun* **135**, 189-195, doi:10.1016/0006-291x(86)90961-7 (1986).
2. Krumschnabel, G., Eigentler, A., Fasching, M. & Gnaiger, E. Use of safranin for the assessment of mitochondrial membrane potential by high-resolution respirometry and fluorometry. *Methods Enzymol* **542**, 163-181, doi:10.1016/B978-0-12-416618-9.00009-1 (2014).
3. Ravasz, D. *et al.* Reduction of 2-methoxy-1,4-naphtoquinone by mitochondrially-localized Nqo1 yielding NAD(+) supports substrate-level phosphorylation during respiratory inhibition. *Biochimica et biophysica acta. Bioenergetics* **1859**, 909-924, doi:10.1016/j.bbabio.2018.05.002 (2018).

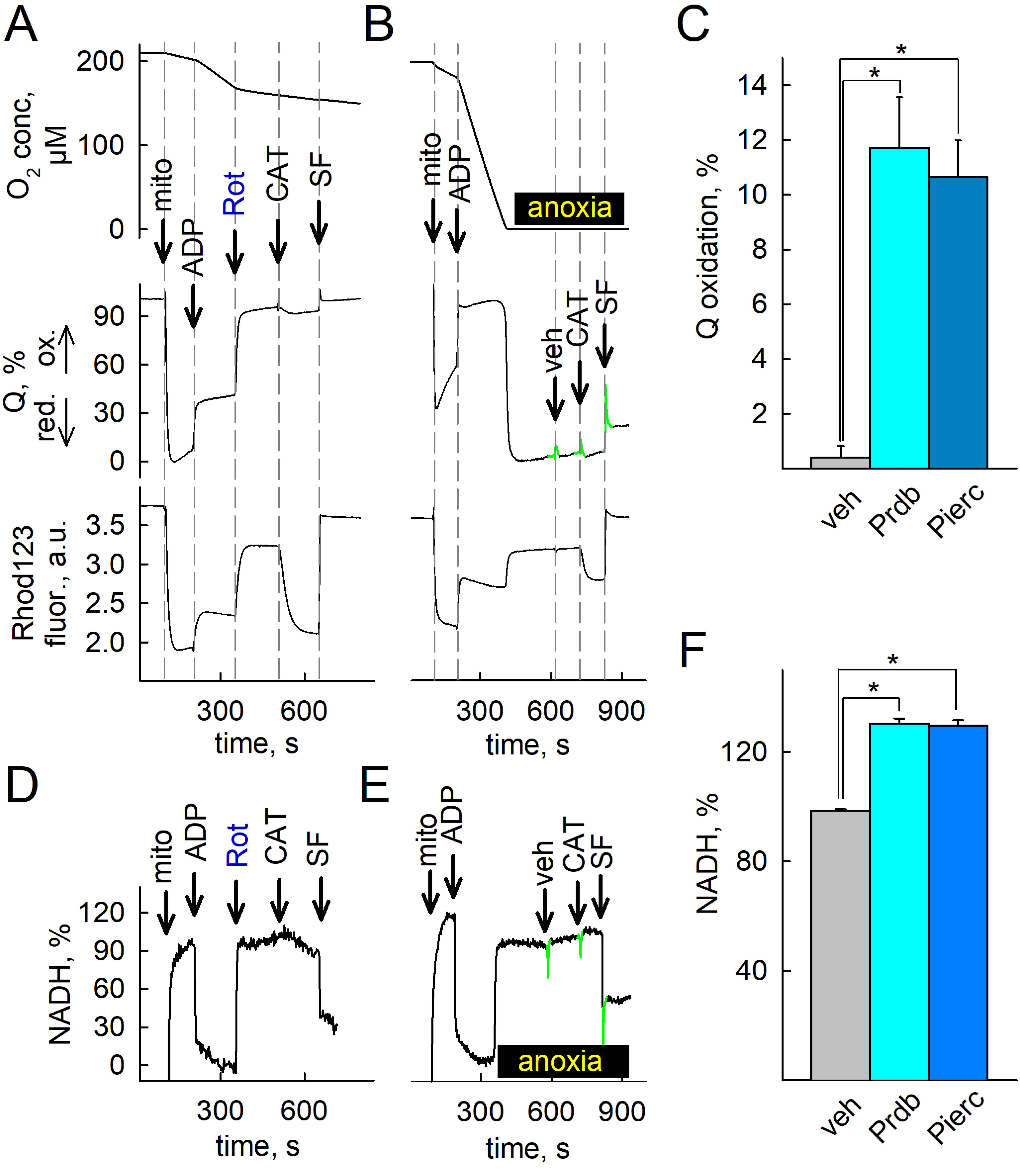

Supplementary Figure 1: oxygen concentration recorded simultaneously with UQ redox state and rhodamine 123 fluorescence in isolated mouse liver mitochondria; quantification of changes in Q and NADH signal in anoxic mitochondria

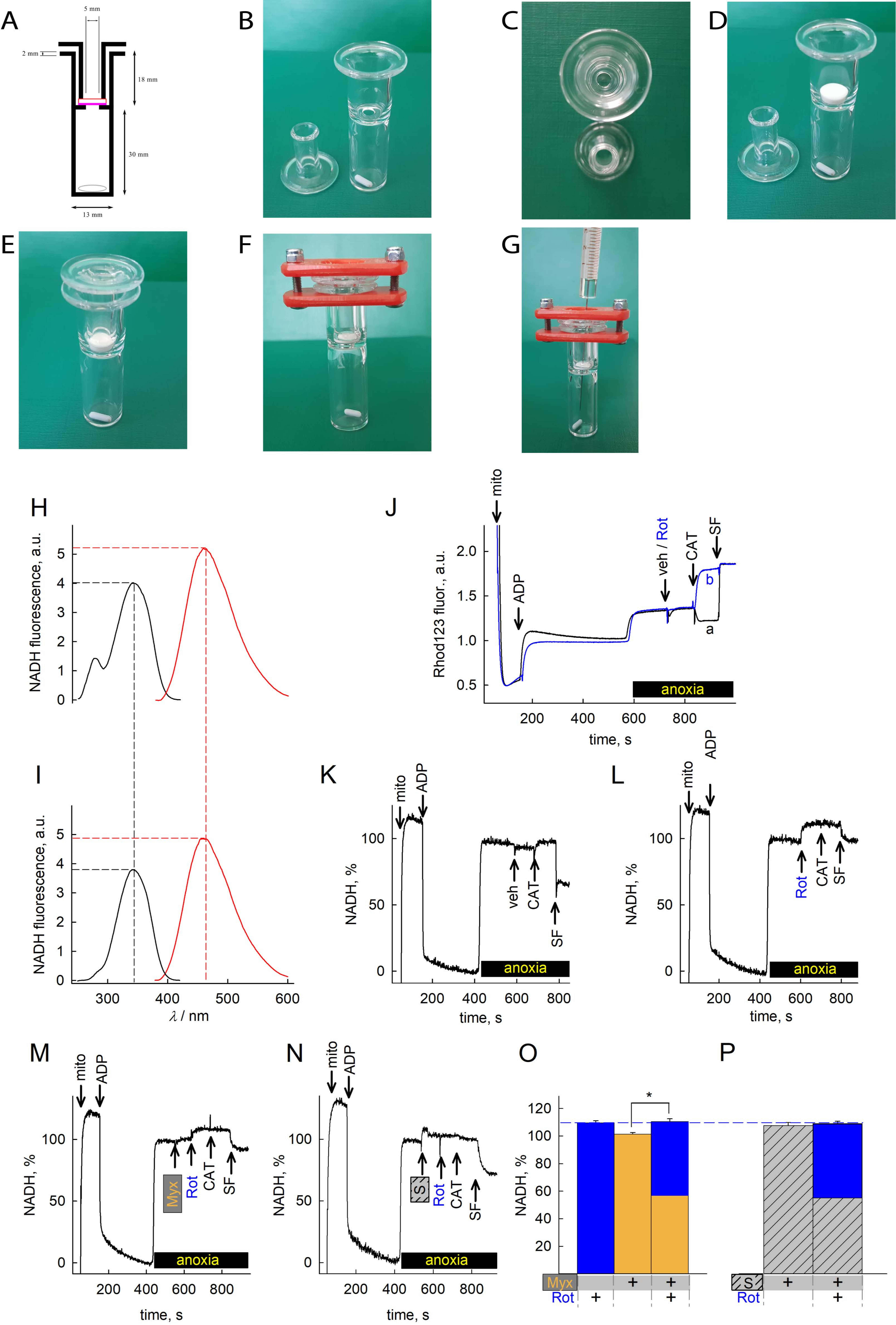

Supplementary figure 2: NADH and Rhod123 fluorescence in anoxic mitochondria suspended in an air-tight glass chamber

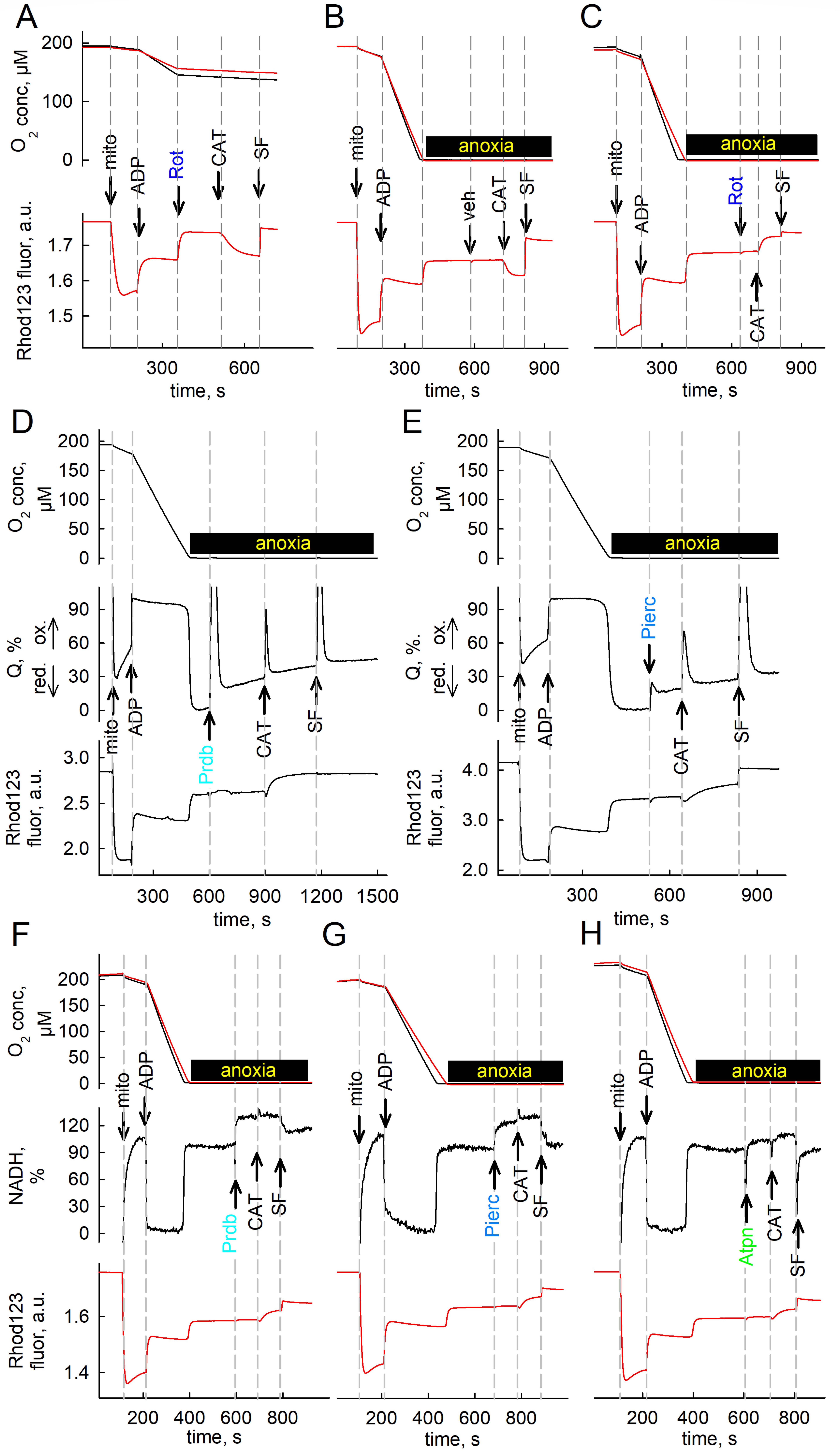

Supplementary Figure 3: oxygen concentration recorded simultaneously with UQ redox state or NADH and rhodamine 123 fluorescence in isolated mouse liver mitochondria

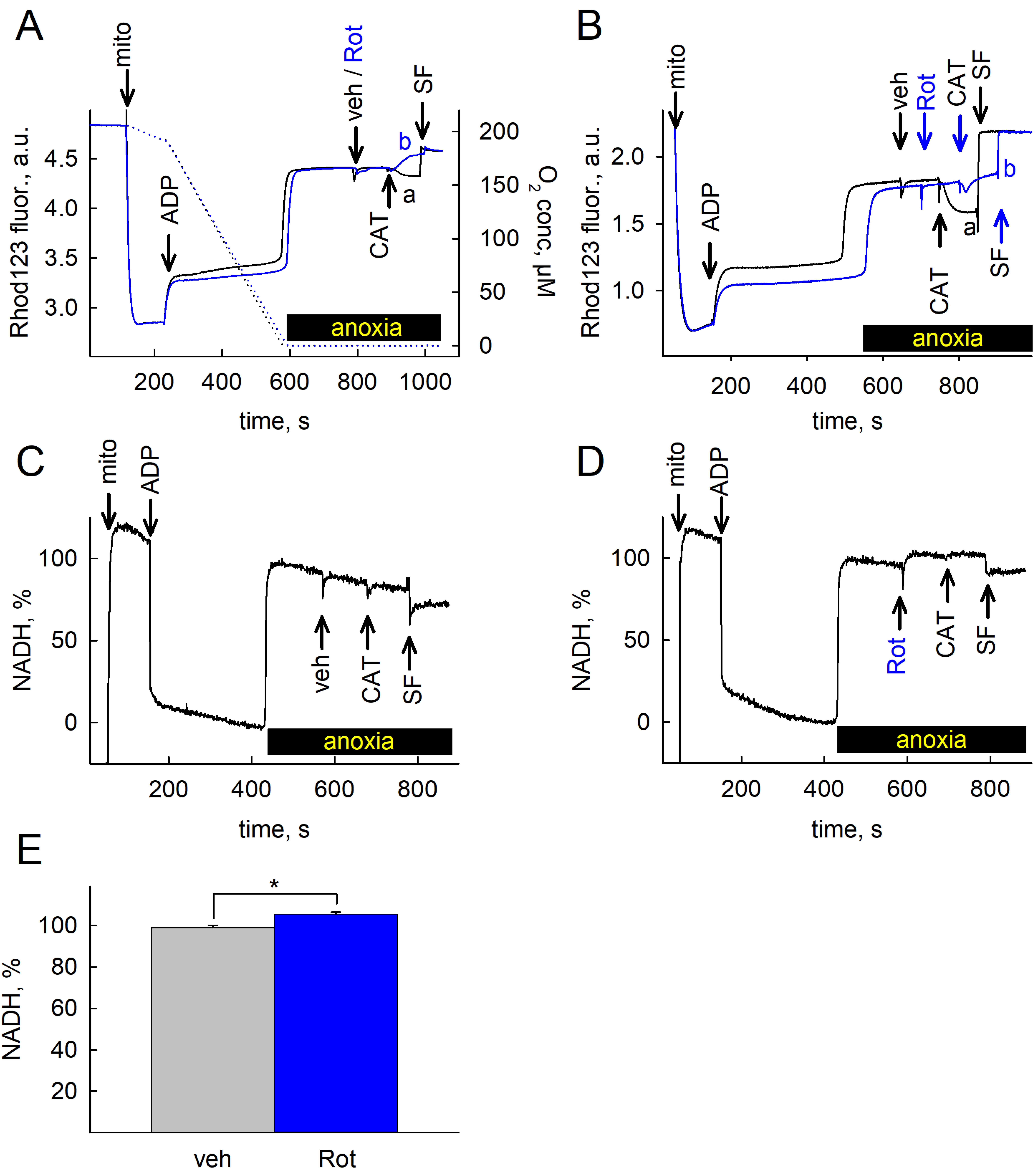

Supplementary figure 4: Residual Complex I activity in anoxic mouse brain mitochondria

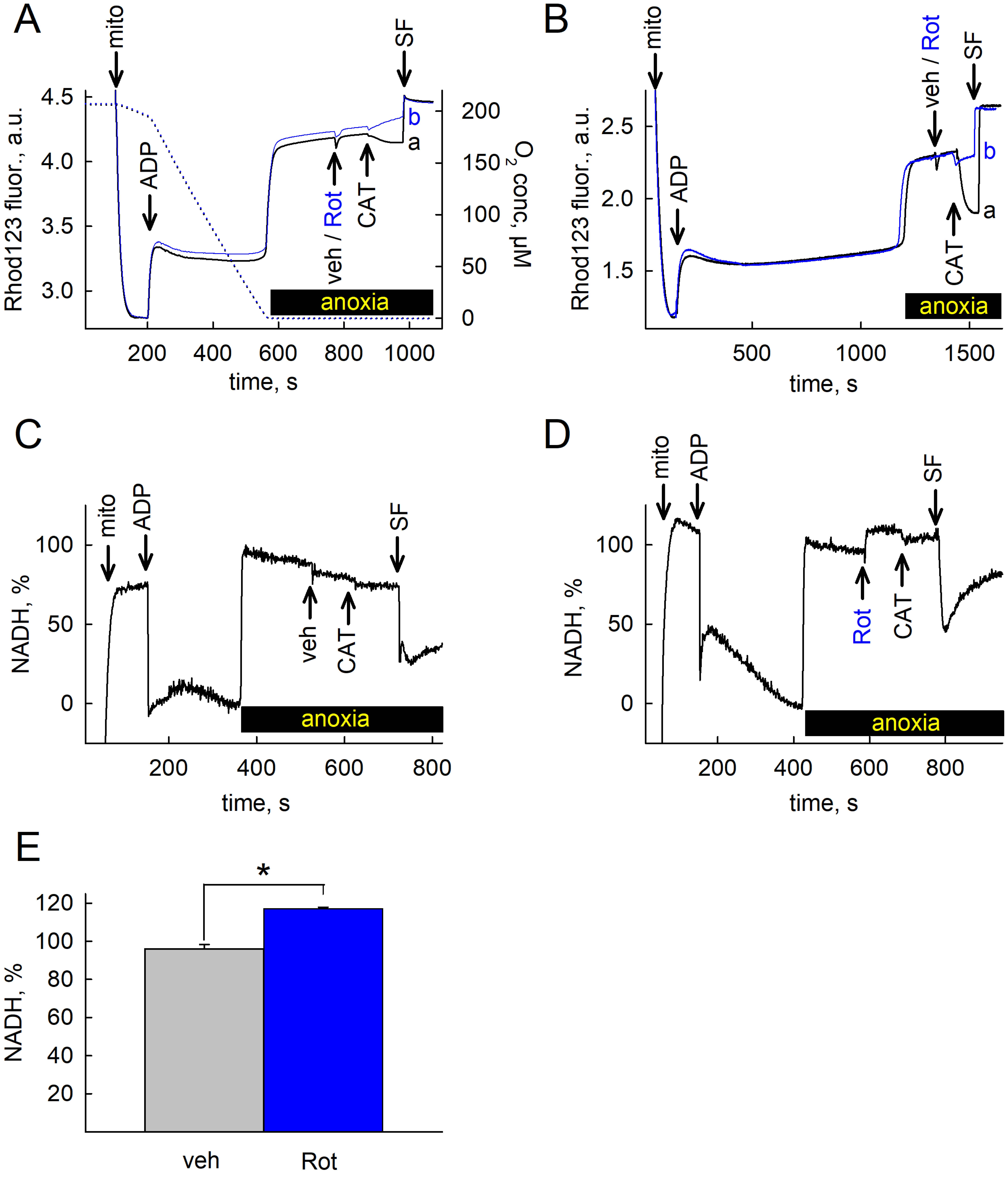

Supplementary figure 5: Residual Complex I activity in anoxic mouse heart mitochondria

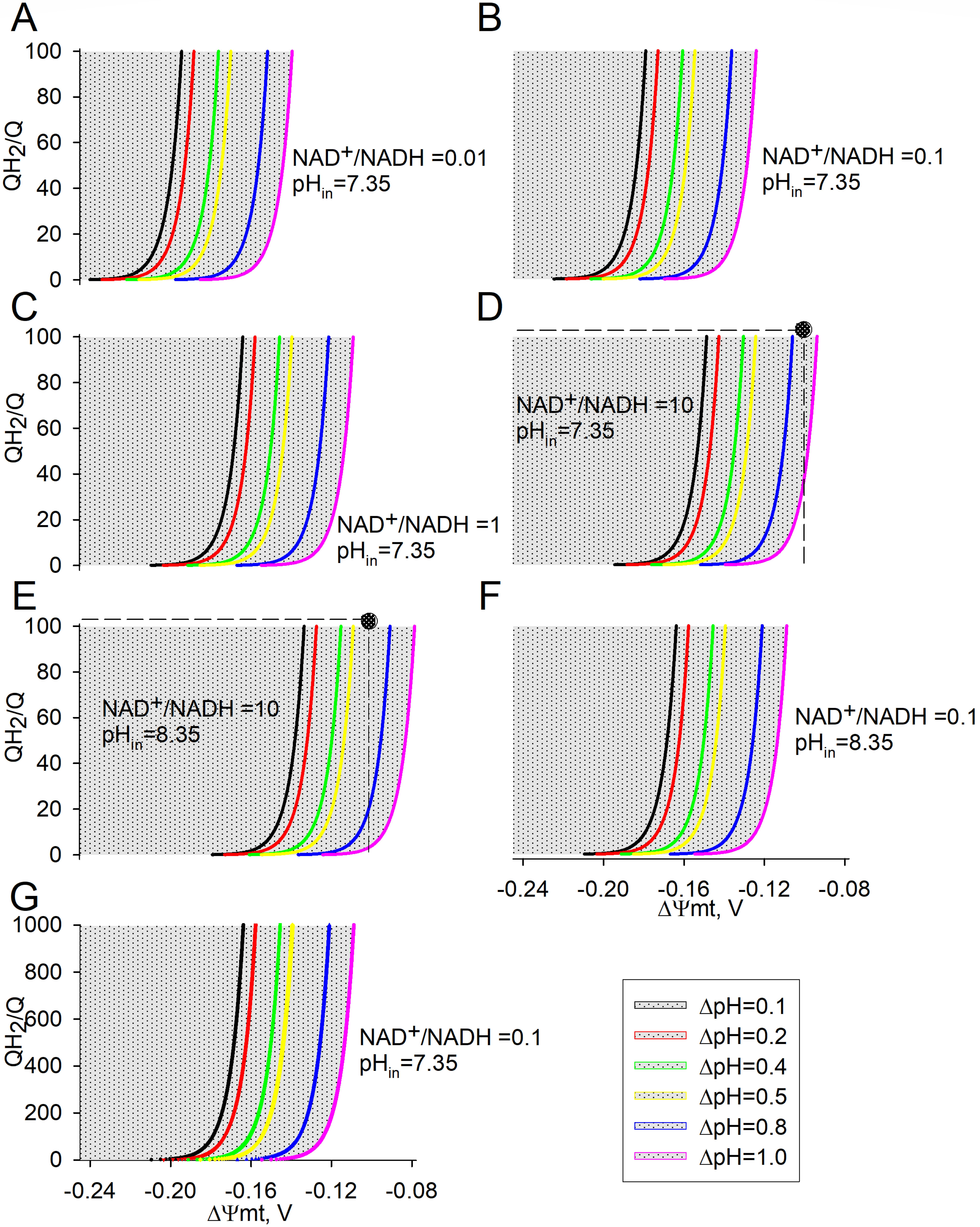

Supplementary figure 6: Directionality of CI operation

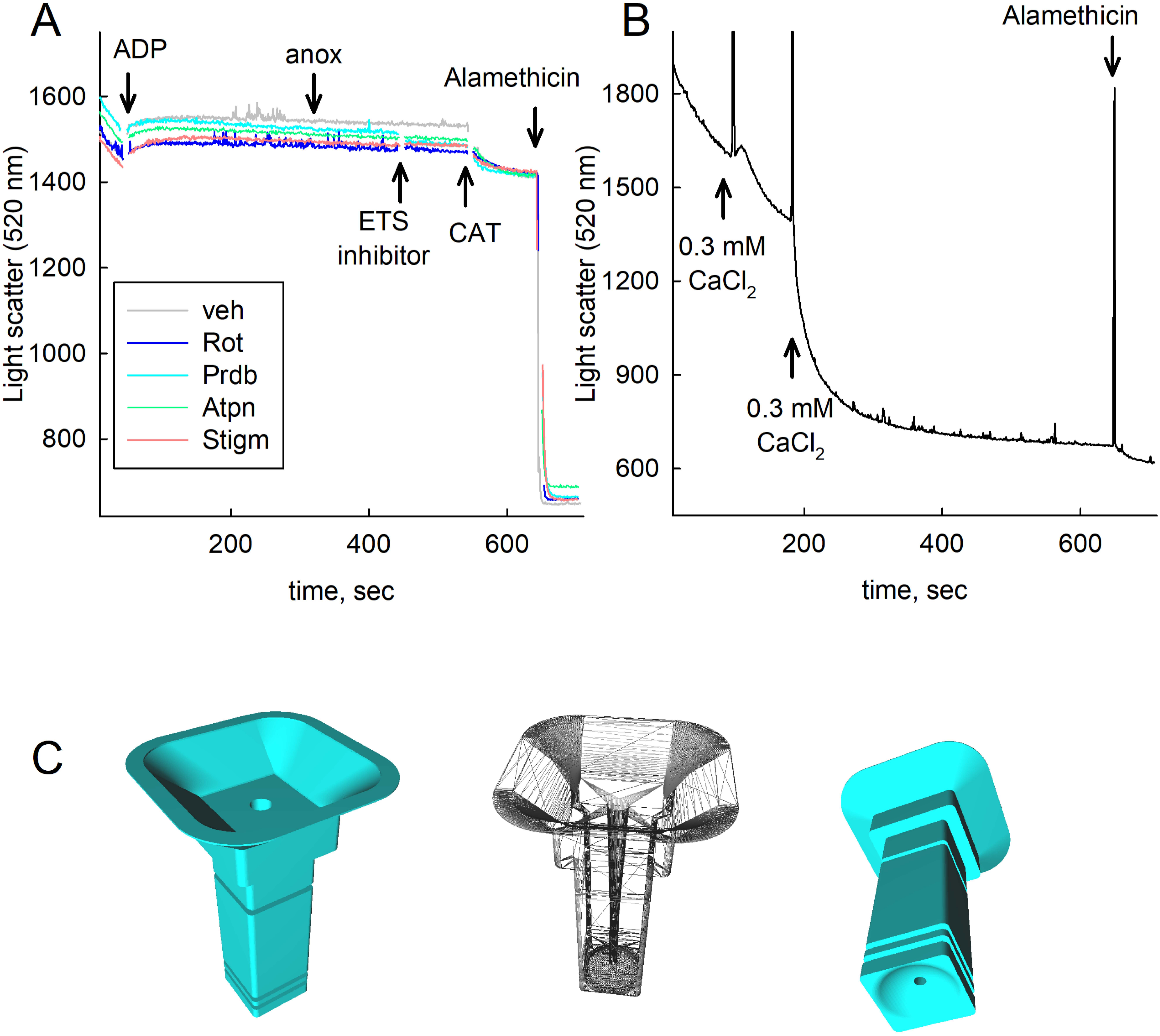

Supplementary figure 7: Light scatter of anoxic mouse liver mitochondria

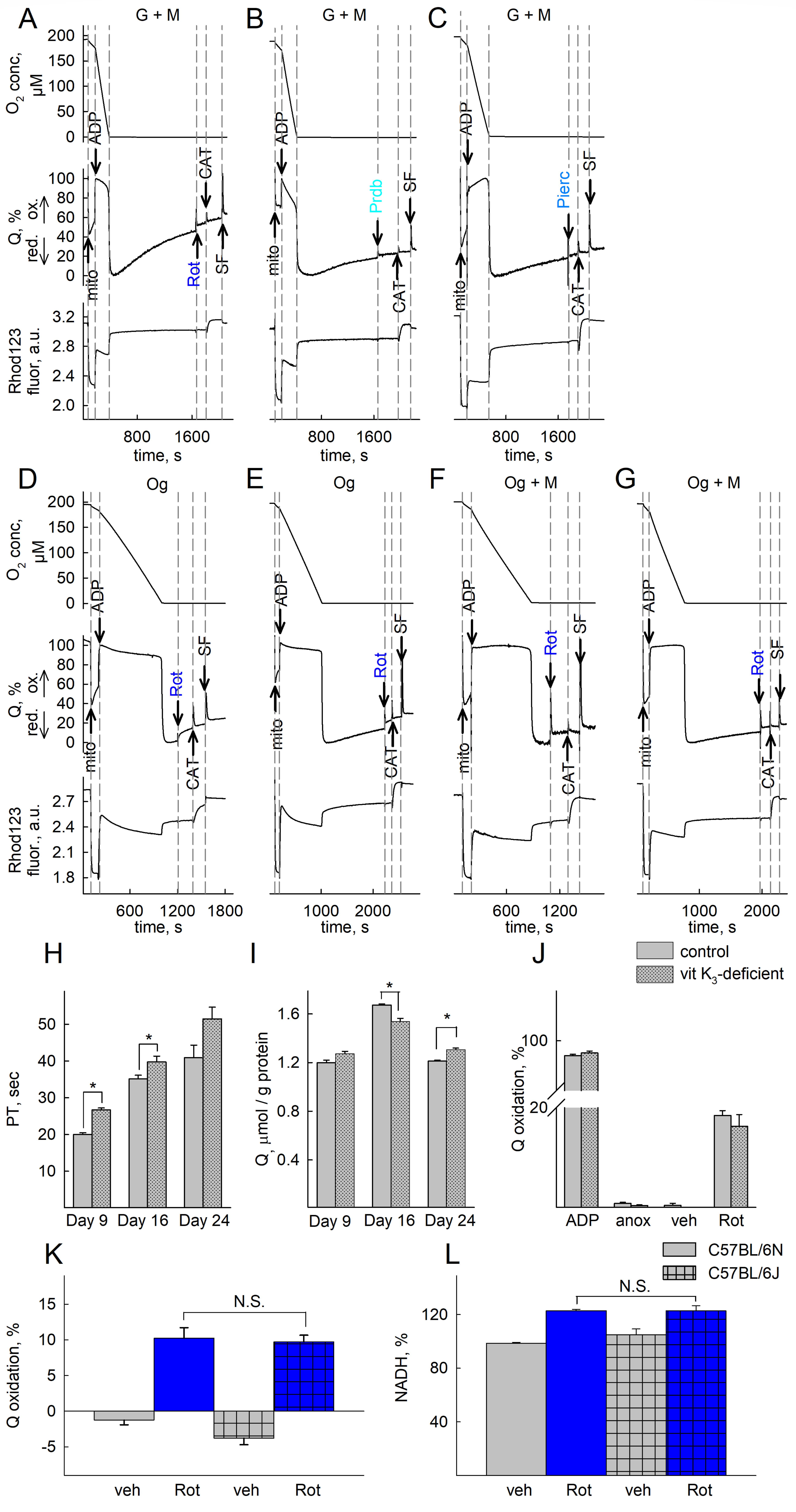

Supplementary figure 8: Representative traces of CI-inhibitor-induced changes in Q signal, and effect of vit K supplementation and NNT

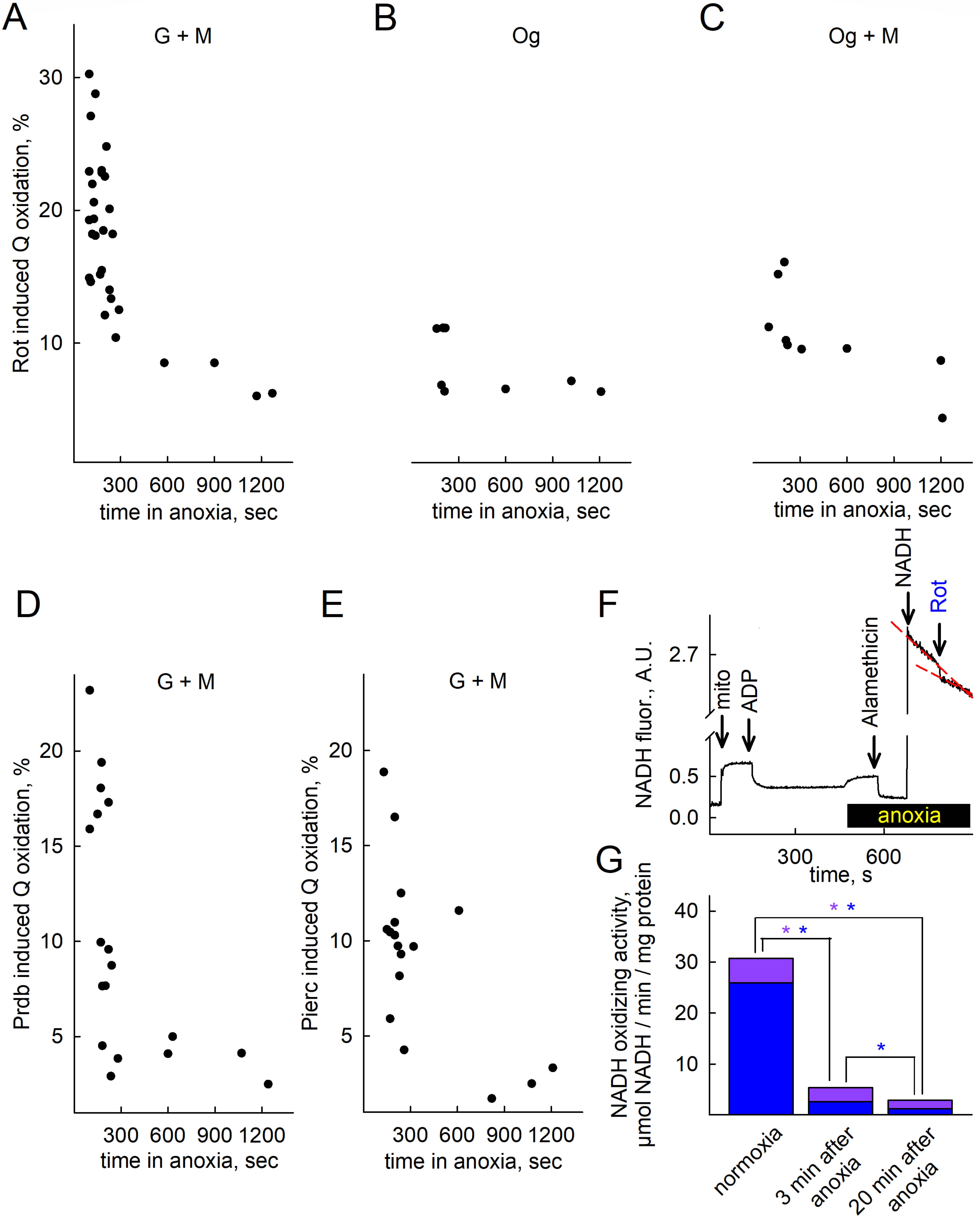

Supplementary figure 9: The CI inhibitor-induced change in UQ signal (% scale) plotted as a function of time elapsed from the onset of anoxia until the addition of CI inhibitors

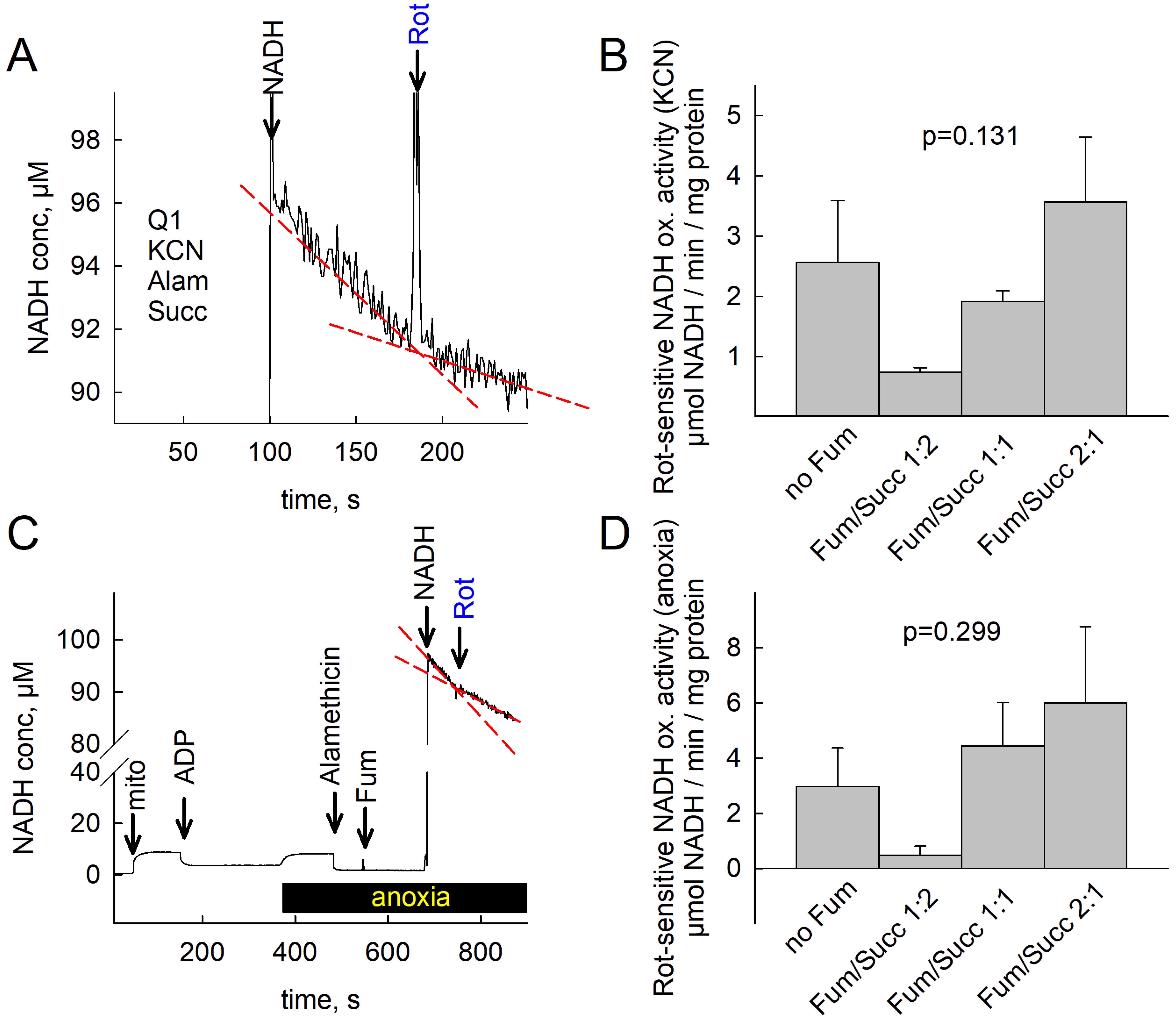

Supplementary figure 10: Fumarate reductase activity in anoxic mitochondria as a function of Fum/Succ ratio

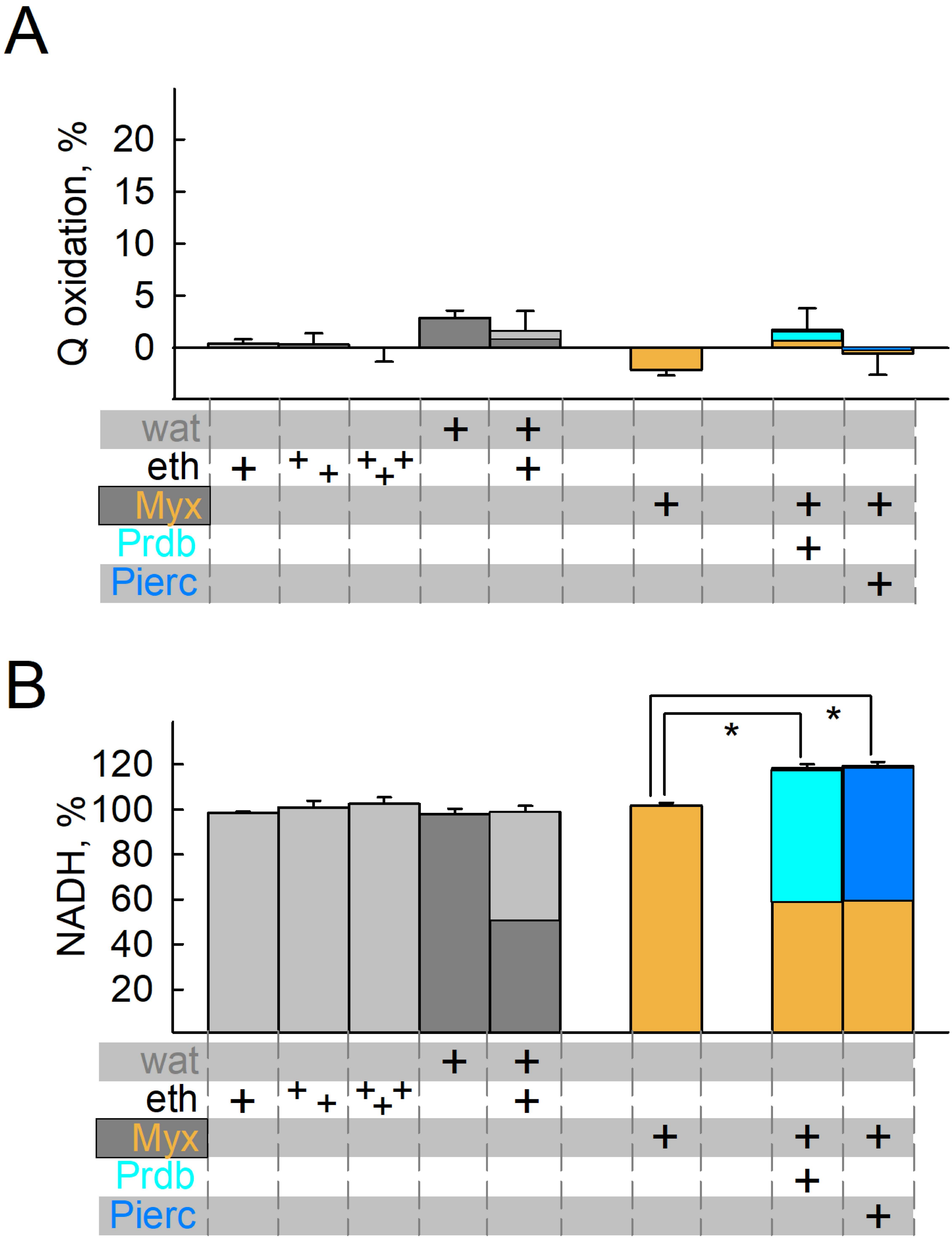

Supplementary figure 11: Quantification of inhibitor-induced changes in Q or NADH

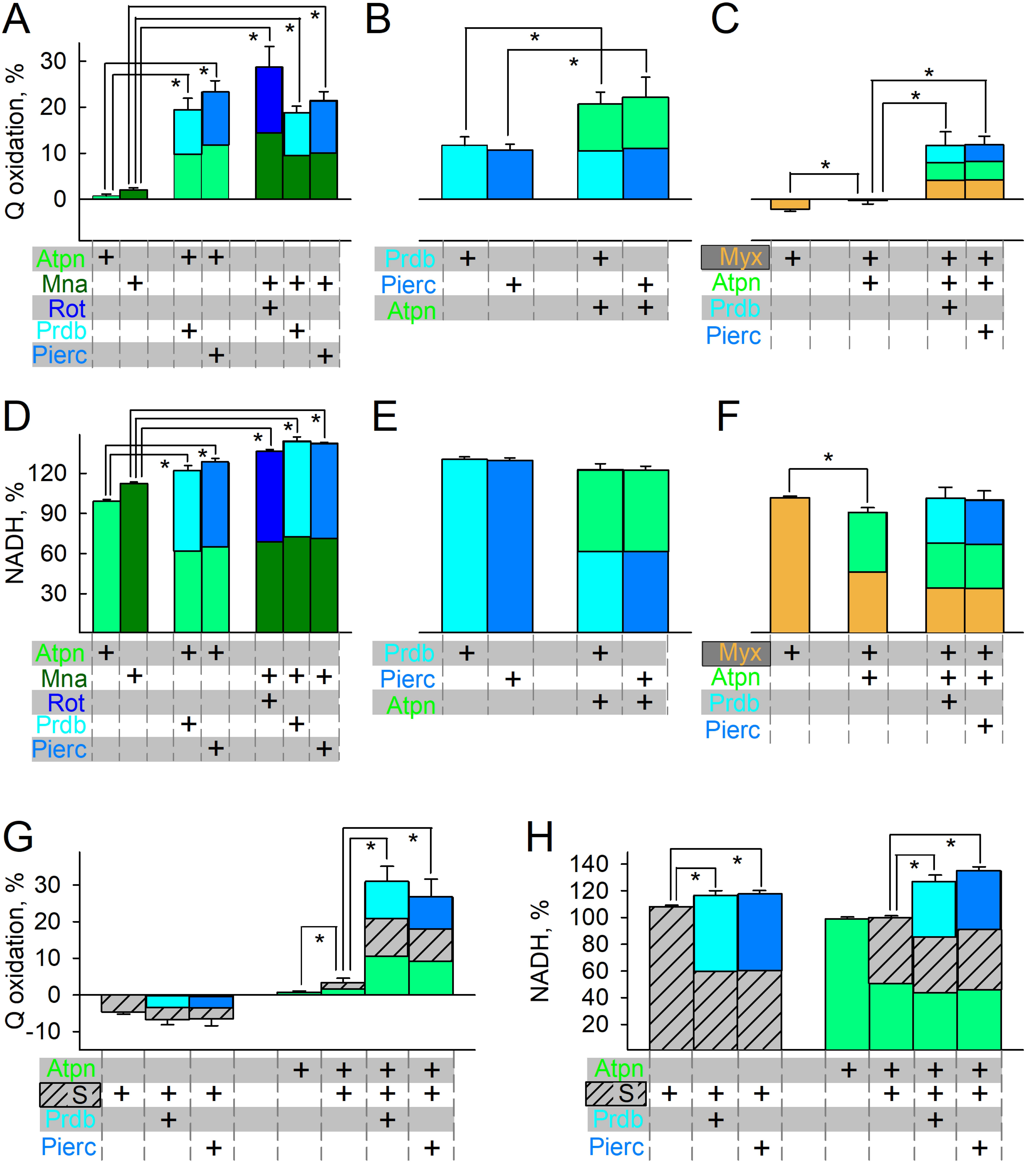

Supplementary figure 12: Quantification of inhibitor-induced changes in Q or NADH using different inhibitor combinations from those in suppl figure 11.

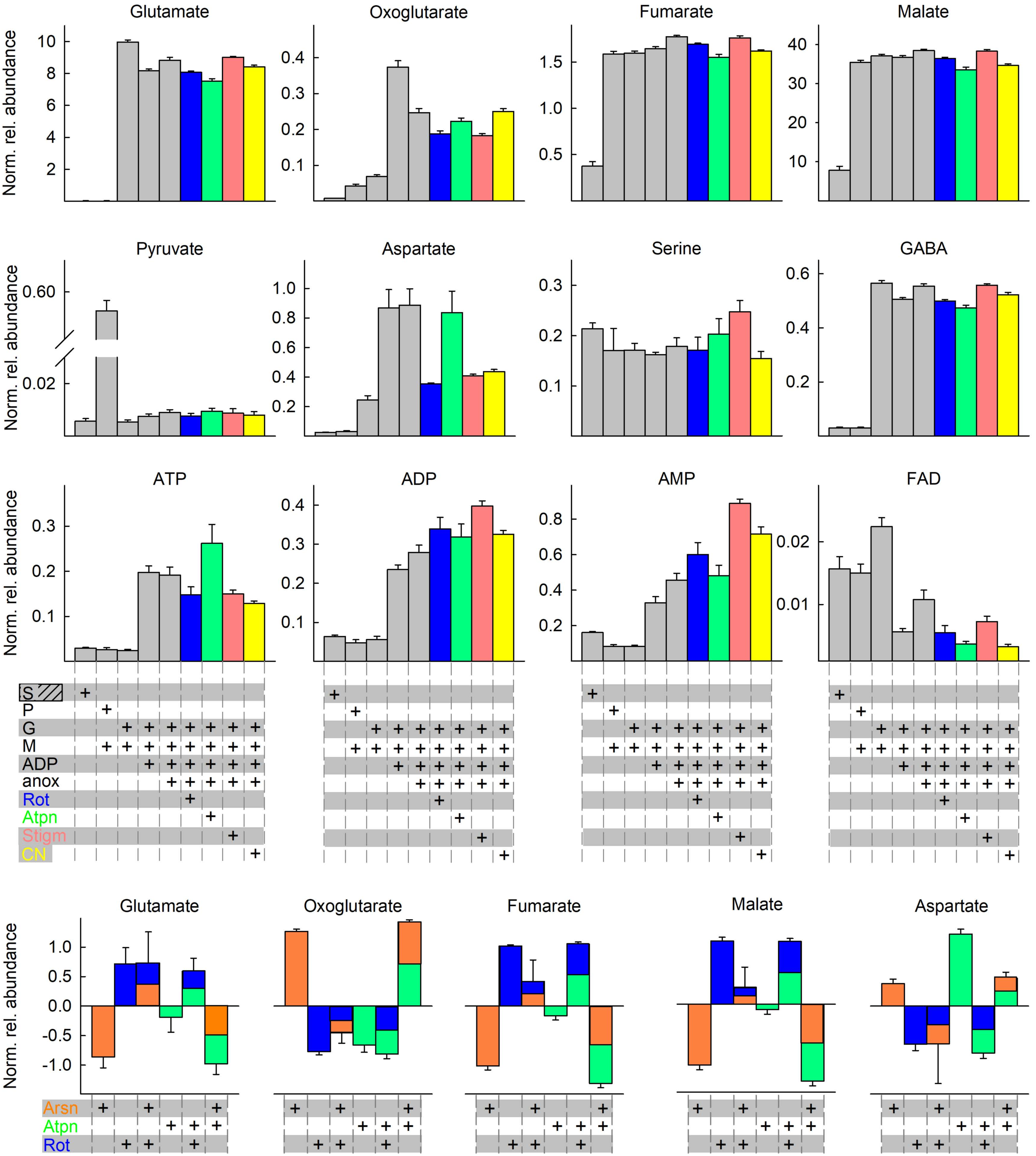

Supplementary figure 13: Analysis of untargeted metabolites present in the pellets of mitochondria treated with the conditions indicated in the panel

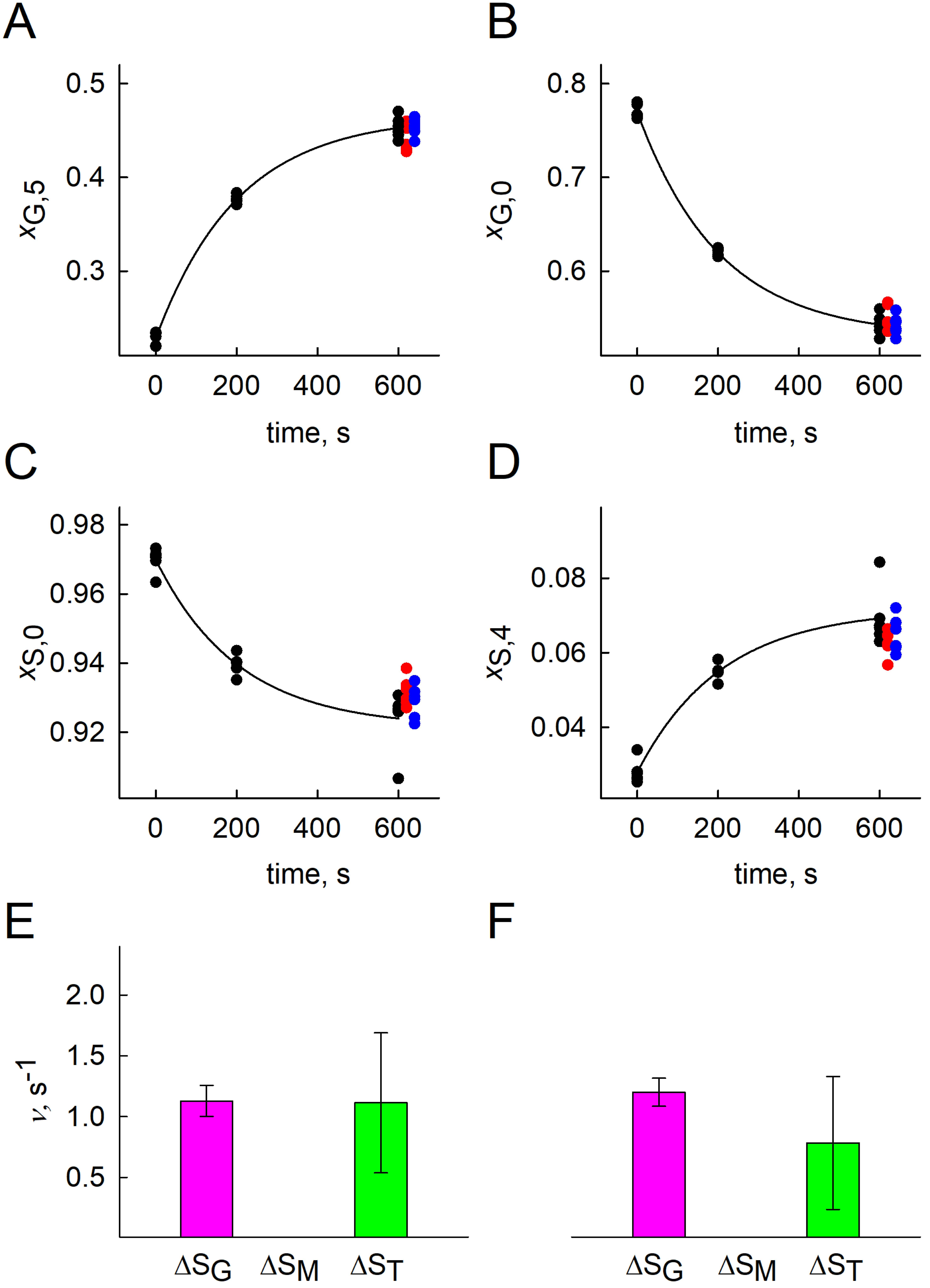

Supplementary figure 14: Changes in isotopologue composition over time

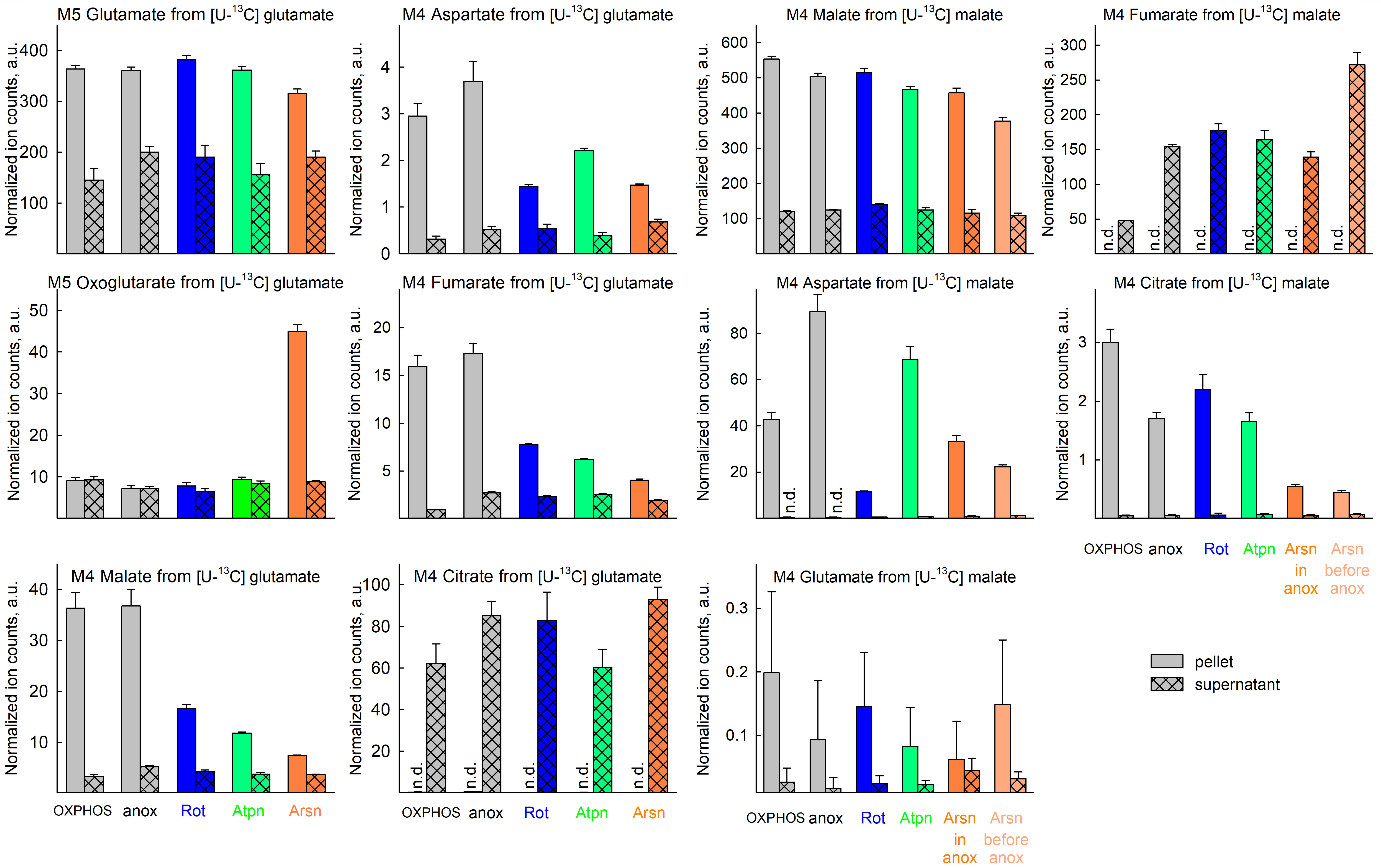

Supplementary figure 15: 13C-tracer analysis of metabolites present in the effluxes (supernatants) or pellets of mitochondria treated with the conditions indicated in the panels

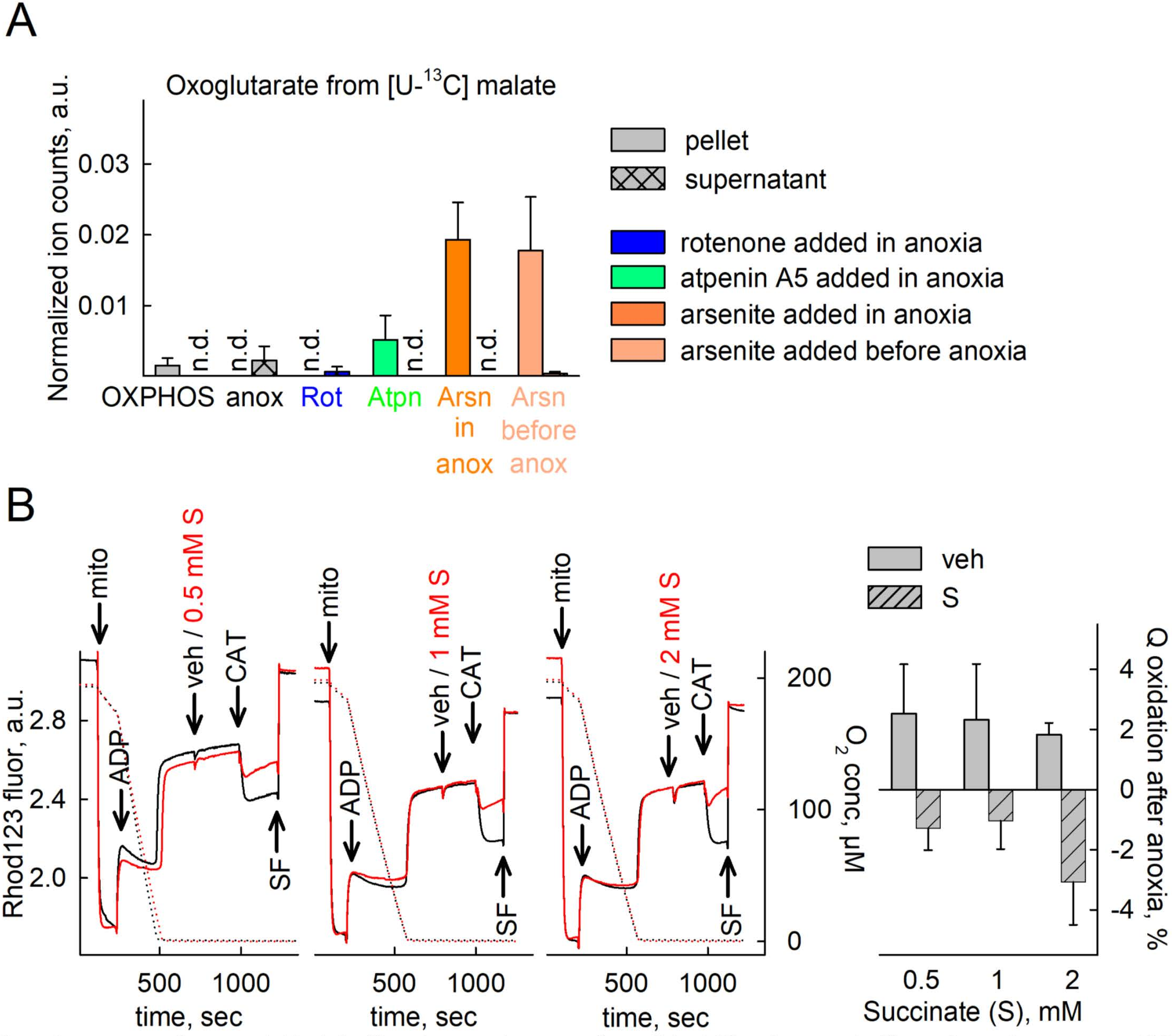

Supplementary figure 16: No labelling in oxoglutarate from [U-13C]malate and effect of succinate on mtSLP

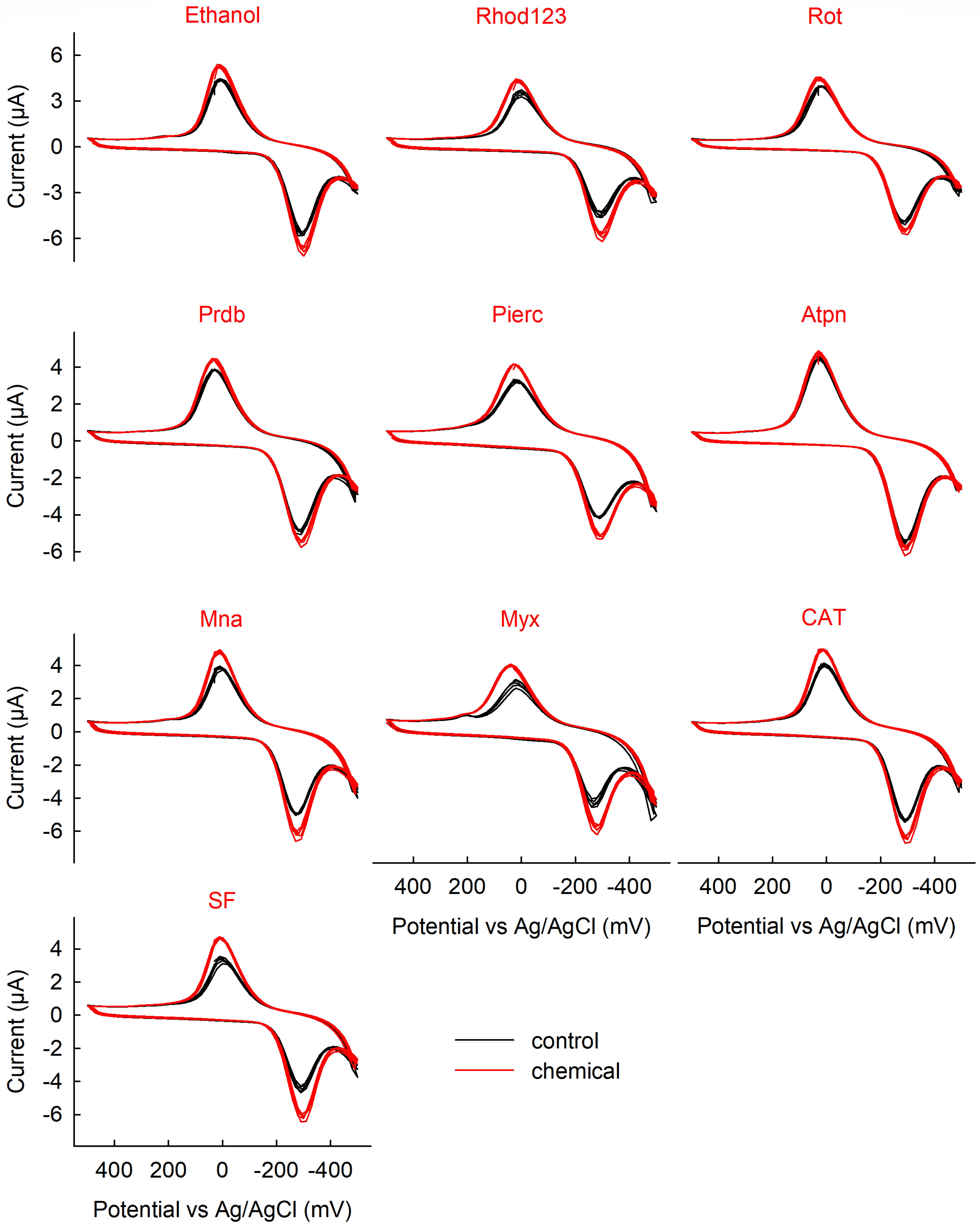

Supplementary figure 17: Cyclic voltammograms in the absence (black traces) or presence (red traces) of chemicals used in this study

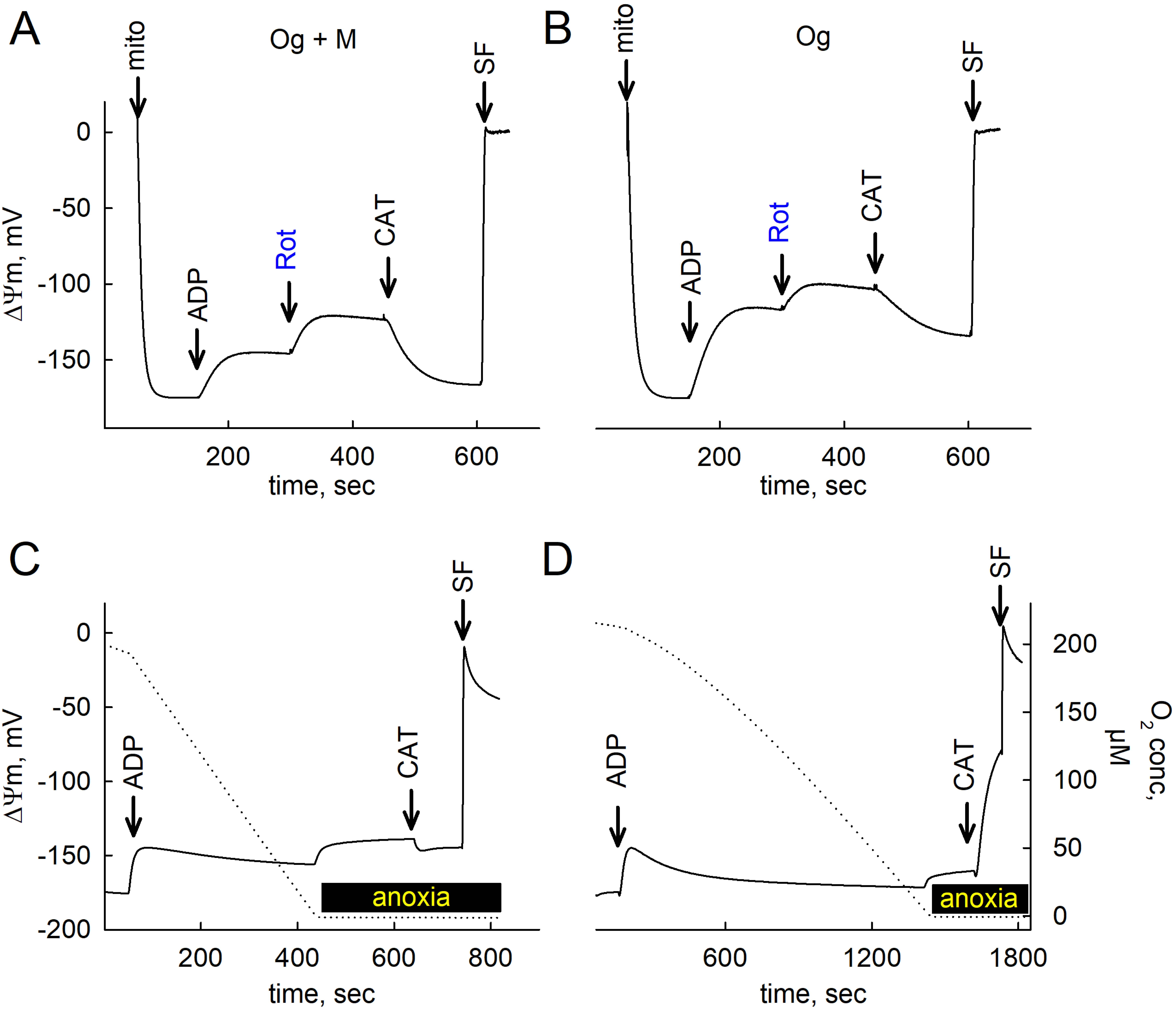

Supplementary figure 18: mtSLP in CI-inhibited using rotenone (Rot) (A, B) vs anoxic (C, D) mitochondria as a function of either Og+malate (A, C) or only Og (B, D). Reproduced by permission from reference 3 (reference list of supplementary text)
